# Causes of hospitalization among people living with HIV in a tertiary-care hospital in Bogotá, Colombia, 2018–2024

**DOI:** 10.1371/journal.pone.0357788

**Published:** 2026-09-30

**Authors:** Daniela Lucía Rico-Bolívar, Cándida Díaz-Brochero, Rebecca Hattemer, Camila Andrea Bejarano-Mora, Laura Bejarano-Mora, Moises A. Huaman, Sandra Liliana Valderrama-Beltrán

**Affiliations:** 1 Pontificia Universidad Javeriana, Faculty of Medicine, Department of Internal Medicine, Bogotá, Colombia; 2 Division of Infectious Diseases, Department of Internal Medicine, Hospital Universitario San Ignacio, Bogotá, Colombia; 3 Department of Clinical Epidemiology and Biostatistics, Pontificia Universidad Javeriana, Bogotá, Colombia; 4 University of Cincinnati College of Medicine, Cincinnati, Ohio, United States of America; 5 Division of Infectious Diseases, Department of Internal Medicine, University of Cincinnati College of Medicine, Cincinnati, Ohio, United States of America; Gulu University, UGANDA

## Abstract

**Background and objective:**

Despite expanded access to antiretroviral therapy (ART), people living with HIV (PLWH) continue to experience high hospitalization rates, particularly in low- and middle-income countries. This study aimed to describe the causes of hospitalization in a cohort of PLWH treated at a high-complexity referral center in Bogotá, Colombia, and to assess variations across the pre-pandemic, pandemic, and post-pandemic periods of COVID-19.

**Methodology:**

We conducted a retrospective cohort study including hospitalization episodes among adults with confirmed HIV infection between 2018 and 2024. Clinical characteristics, causes of hospitalization, and outcomes were compared across the epidemiological periods related to the COVID-19 pandemic.

**Results:**

A total of 872 hospitalization episodes corresponding to 584 patients were analyzed. The median age was 34 years, and 89.1% were men. Although 68.7% of patients had a history of ART use, 54.8% were admitted with detectable viral load. Non–AIDS-defining causes predominated across all periods (61.8%), with a progressive increase from the pre-pandemic to the post-pandemic period (55.3%, 63.1%, and 70.1%, respectively). A sustained increase in hospitalizations due to mental health disorders was observed over time (4.3%, 4.6%, and 7.8%). Among AIDS-defining conditions, tuberculosis (15.8%) and *Pneumocystis jirovecii* pneumonia (14.9%) were the most frequent. Hospitalizations due to opportunistic infections were associated with longer length of stay (median 14 vs. 6 days; p < 0.001) and higher 30-day mortality (8.6% vs. 2.6%; p < 0.001) compared with non–AIDS-defining hospitalizations, with a proportional increase in events attributable to tuberculosis and Kaposi sarcoma.

**Conclusions:**

An epidemiological transition towards non-AIDS-defining causes of hospitalization is evident, comparable to patterns observed in high-income countries; however, a substantial burden of tuberculosis, Kaposi sarcoma, and late HIV diagnosis persists, likely reflecting ongoing structural gaps in HIV care. Following the COVID-19 pandemic, an increase in hospitalizations related to mental health disorders was observed. Taken together, these findings highlight the urgent need to strengthen early diagnosis, active case finding, and tuberculosis prevention, as well as to enhance the implementation of comprehensive care models that improve retention in care for PLWH in low- and middle-income countries.

## Introduction

The human immunodeficiency virus (HIV), first identified in 1983 [1], has caused approximately 44.1 million deaths worldwide and has remained a global public health challenge for more than four decades [2]. Since its introduction, antiretroviral therapy (ART) has advanced rapidly, and access to these drugs has increased exponentially. The United Nations’ goal is to end the AIDS pandemic by 2030 through the 95-95-95 strategy (diagnosing at least 95% of people living with HIV (PLWH), ensuring that 95% of them receive ART, and achieving sustained viral suppression in 95% of those on treatment) [3].

As of 2024, an estimated 40.8 million people were living with HIV globally; of these, 31.6 million were receiving ART, compared to the 7.7 million reported in 2010 [4]. This expansion in treatment coverage has resulted in a significant decline in HIV-related morbidity and mortality. Cohorts from high-income countries have documented a reduction in opportunistic infections and AIDS-defining illnesses, alongside a relative increase in non-AIDS-defining comorbidities associated with population aging and the chronic nature of the infection [5–7].

A recent meta-analysis of hospital admissions among PLWH, covering the period 2014–2023, confirmed that AIDS-related diseases remain a predominant cause of hospitalization worldwide, particularly in resource-limited regions [8]. However, the distribution of admission causes is heterogeneous: in Europe and the United States, non-AIDS-defining illnesses—such as cardiovascular disease, non-AIDS-defining malignancies, and metabolic complications—predominate. Conversely, in low- and middle-income countries, opportunistic infections and AIDS-defining illnesses remain the leading causes of hospitalization, accounting for nearly 62% of admissions in Latin America [8].

In Colombia, national surveillance data identified 185,954 people living with HIV in 2024, corresponding to a prevalence of approximately 356 per 100,000 inhabitants. During the same year, 14,555 new cases were reported, with an adjusted incidence of 26.90 per 100,000 inhabitants and an adjusted all-cause mortality rate of 4.01 per 100,000 inhabitants; both indicators declined compared with the previous year [9]. Antiretroviral therapy (ART) coverage reached 80.74% of registered cases, and 89.42% of those receiving treatment achieved viral suppression [9]. Despite the availability of these surveillance indicators, evidence on hospitalization among PLWH in Colombia remains scarce. No national hospitalization rate has been published, and the most comprehensive evidence available comes from a 2017 multicenter study conducted in six hospitals in Medellín, in which infectious diseases—particularly tuberculosis, *Pneumocystis jirovecii* pneumonia, and other opportunistic infections—were the leading causes of hospitalization and death [10]. Recent national evidence regarding hospitalization profiles in the era of widespread ART availability remains limited, especially in high-complexity institutions.

To our knowledge, there are no recent studies evaluating the impact of the COVID-19 pandemic on the causes of hospitalization in Colombia. The objective of this study was to characterize the causes of hospitalization in a cohort of adults with HIV infection treated at a tertiary-care hospital in Bogotá, Colombia, describe their variations according to the epidemiological periods of the COVID-19 pandemic, and discuss these findings in the context of current literature.

## Materials and methods

### Study design and population

We conducted a retrospective observational cohort study at Hospital Universitario San Ignacio (HUSI), a private, not-for-profit university hospital affiliated with the Pontificia Universidad Javeriana in Bogotá, Colombia. Bogotá is the capital and largest city of the country, with a population of approximately 7.9 million in 2024 [11]. Colombia has a near-universal health insurance system that operates through contributory and subsidized schemes, with care delivered by healthcare institutions of varying levels of complexity. As a high-complexity teaching and referral center, HUSI provides specialized medical and surgical care. Its Infectious Diseases service includes a dedicated HIV care program serving people living with HIV affiliated with multiple health insurers (Empresas Promotoras de Salud, EPS), which reinforces HUSI’s role as a referral center for HIV care in Bogotá and surrounding areas. Eligibility was based on HIV status rather than on the reason for admission. We included all hospitalization episodes of adults (≥18 years) with a length of stay >24 hours and a confirmed diagnosis of HIV infection established before admission or during hospitalization, according to international diagnostic guidelines [12]. The study period comprised admissions from January 1, 2018, through June 30, 2024. Episodes in which patients were referred or transferred to another institution within the first 72 hours of admission were excluded because complete characterization of the clinical event and its outcomes could not be ensured.

The unit of analysis was the hospitalization episode. To promote independence of events, readmissions were not considered new episodes when they occurred within 30 days of discharge and corresponded to the same primary cause, or at any later time if they were related to complications directly attributable to the index event. In contrast, readmissions for a different primary cause were considered new independent hospitalization episodes, regardless of the time elapsed since discharge.

### Data processing and collection

Patients were identified by querying an institutional database of all HUSI hospitalizations using a broad set of infectious-disease ICD-10 codes. This case-finding strategy intentionally included diagnoses beyond HIV and therefore retrieved records that did not correspond to confirmed HIV infection. Retrieved records were manually reviewed to confirm HIV infection, and records that did not correspond to confirmed HIV infection were excluded. Eligibility was then verified by cross-referencing the Infectious Diseases service database, which also identified additional eligible hospitalization episodes not captured through administrative coding.

Clinical and sociodemographic data were extracted from the electronic medical records by trained members of the research team, including physicians and residents, and entered into a structured REDCap (Research Electronic Data Capture) database [13,14]. Data collection was standardized using a predefined instrument with controlled response options and built-in field-validation rules. As a quality-control measure, a sample of records was independently cross-checked against the source medical records by a second reviewer, and any identified discrepancies were corrected by returning to the primary source. All sociodemographic variables, including educational level and sexual orientation, were obtained exclusively from the electronic medical records; no direct patient interviews were conducted for this study. These variables had been documented during routine clinical care by members of the interdisciplinary care team, including physicians, nursing staff, and social workers. Variables not documented in the medical record were treated as missing.

### Study variables

Sociodemographic variables included age, sex, sexual orientation, and educational level. Sexual orientation was abstracted as documented during routine clinical care and categorized as heterosexual, homosexual, bisexual, or not documented. Educational level was categorized as primary, secondary, technical, university/professional, or not documented, and was used as a proxy for socioeconomic position. Psychoactive substance use was based primarily on patient self-report documented during routine clinical care, including the structured psychosocial assessment performed by the social work team, and was subsequently abstracted from the electronic medical record. No study-specific interviews or toxicological testing were performed. Substance use was recorded as a multiple-response variable using predefined categories: intravenous drug use, inhaled drug use, other drug use, and active cigarette smoking. Clinical variables included timing of HIV diagnosis, clinical stage according to the Centers for Disease Control and Prevention (CDC), baseline immune status at diagnosis, comorbidities, and hepatitis B virus (HBV) or hepatitis C virus (HCV) coinfection. Admission laboratory values were recorded to characterize immunovirological status (CD4 cell count and HIV viral load). Outcomes included length of hospital stay, ICU admission, 30-day readmission, in-hospital mortality, and 30-day mortality.

Causes of hospitalization were classified into two main groups: AIDS-defining conditions and non–AIDS-defining conditions. The latter included the following subcategories: non-opportunistic infections, surgical conditions, neurologic conditions, gastrointestinal conditions, cardiovascular conditions, non–AIDS-related neoplasms, pulmonary conditions, genitourinary conditions, autoimmune conditions, trauma-related conditions, mental health conditions, endocrine conditions, renal conditions, adverse drug reactions, and other causes.

### Determination of the cause of hospitalization

For each episode, diagnoses directly related to the reason for admission were identified and recorded as the main cause(s) of hospitalization. Classification was based on the clinical judgment of the treating team verified by the research investigators. When two conditions contributed equally to the admission, both were recorded as co-primary causes, allowing more than one primary etiology per episode. Clinically relevant diagnoses identified during hospitalization that were not the initial reason for admission (e.g., nosocomial infections or decompensation of comorbidities) were recorded as secondary causes.

### Operational definitions

Causes of hospitalization were grouped into AIDS-defining conditions, based on the CDC definition [15], and non–AIDS-defining conditions, defined as any primary diagnosis not included in the AIDS-defining category [16,17]. HIV clinical stage was categorized according to the current CDC classification [18].

Adherence to antiretroviral therapy (ART) was assessed by patient self-report elicited by the treating clinical team during routine care and documented in the electronic medical record; this information was subsequently abstracted by the research team, and no adherence interviews were conducted specifically for this study. For the purposes of this study, optimal adherence was defined as taking ≥95% of prescribed doses in the prior month [19,20]. Adherence below this threshold was classified as suboptimal and included behaviors ranging from intermittent missed doses to treatment interruption. Treatment interruption was further categorized as short-term (<1 month) or prolonged (≥1 month).

For outcome assessment, 30-day readmission was defined as any inpatient readmission within 30 days of discharge, regardless of whether the cause was the same as or different from that of the index episode, consistent with prior studies in people with HIV [21].

To examine reasons for hospitalization across the COVID-19 context, admissions were stratified into three epidemiological periods based on milestones declared by the World Health Organization (WHO) and the declaration of a health emergency in Colombia [22]: (1) pre-pandemic, admissions before March 1, 2020; (2) pandemic, admissions from March 1, 2020, to May 5, 2023; and (3) post-pandemic, admissions after May 5, 2023.

### Statistical analysis

We performed descriptive analyses of sociodemographic and clinical characteristics and outcomes. Normality of continuous variables was assessed using the Shapiro–Wilk test. Data are presented as mean (standard deviation, SD) for normally distributed variables and median (interquartile range, IQR) for non-normally distributed variables. Categorical variables are reported as counts and proportions (n, %). Missing data were not imputed; analyses were performed using available cases for each variable, and the corresponding analytic denominator is reported in the tables. Variables conditional by design were analyzed within their applicable subgroups and were not considered missing when not applicable. Values recorded as unknown or not documented were retained as separate categories in descriptive analyses. The extent of missing data for individual variables is reported in the corresponding tables.

Mortality outcomes included in-hospital mortality and 30-day mortality. For patients discharged before day 30, survival status was verified using the General Social Security Health System affiliation database (ADRES/BDUA) from the Colombian Ministry of Health and Social Protection [23].

Group comparisons (AIDS-defining vs non–AIDS-defining causes) and comparisons across pandemic periods were conducted using chi-square or Fisher’s exact tests for categorical variables and Student’s t test or the Mann–Whitney U test for continuous variables, as appropriate. A two-sided p value <0.05 was considered statistically significant. Results were displayed using bar charts and line graphs. Data processing and statistical analyses were performed in R, version 4.4.2.

### Ethical considerations

The study was conducted in accordance with the Declaration of Helsinki and was approved by the Institutional Research and Ethics Committee of Hospital Universitario San Ignacio (approval No. FM-CIE-0316–25). Given the retrospective design based on medical record review, the study was classified as “research without risk” under local regulations and was therefore exempt from informed consent. Medical records were accessed for research purposes between August 15, 2025, and November 30, 2025. Only the authors involved in data collection had access to information that could identify individual participants during this process; however, all data were anonymized by assigning unique, non-traceable codes to each record, ensuring confidentiality during analysis.

## Results

### Sociodemographic and clinical characteristics

A total of 2,109 records from the hospital administrative database were reviewed. Of these, 857 corresponded to individuals with confirmed HIV infection, whereas 1,252 did not and were excluded after review. Among the 857 records with confirmed HIV infection, 108 episodes were excluded (70 because patients were transferred to another institution within the first 72 hours and 38 because they were readmissions due to the same etiology). An additional 123 eligible hospitalization episodes of people living with HIV, identified through the Infectious Diseases service database but not captured by administrative coding, were included, yielding a final analytic sample of 872 hospitalization episodes among 584 individuals with confirmed HIV infection. The case-identification and inclusion process is summarized in S3 Fig. Characteristics are reported at the episode level (n = 872), as individuals could contribute more than one hospitalization (Table 1).

**Table 1:** Baseline characteristics and outcomes of people living with HIV hospitalized at HUSI, Bogotá, Colombia, between 2018 and 2024, stratified by cause of admission (n = 872).

Characteristics	Total (n = 872)	AIDS defining illness n= 333 (38.2%)	Non-AIDS defining illness n=539 (61.8%)	P Value
Male, n (%)	777 (89.1)	284 (85.3)	493 (91.5)	**0.019**
Age, median (IQR)	34.0 (28.0–47.0)	35.0 (29.0–46.0)	33.0 (27.0–48.5)	0.161
Age group (years)				0.237
18- < 30	290 (33.3)	97 (29.1)	193 (35.8)	
30- < 40	255 (29.2)	108 (32.4)	147 (27.3)	
40- < 50	141 (16.2)	64 (19.2)	77 (14.3)	
50- < 60	128 (14.7)	50 (15.0)	78 (14.5)	
60- < 70	40 (4.6)	9 (2.7)	31 (5.8)	
≥70	18 (2.1)	5 (1.5)	13 (2.4)	
**Sexual orientation, n (%)**				**0.006**
Homosexual	449 (51.5)	149 (44.7)	300 (55.7)	
Heterosexual	291 (33.4)	139 (41.7)	152 (28.2)	
Bisexual	59 (6.8)	23 (6.9)	36 (6.7)	
Unknown	73 (8.4)	22 (6.6)	51 (9.5)	
**Education level, n (%)**				**<0.001**
University/Professional	171 (19.6)	41 (12.3)	130 (24.1)	
Technical	174 (20.0)	58 (17.4)	116 (21.5)	
High school	357 (40.9)	161 (48.3)	196 (36.4)	
Primary education	142 (16.3)	67 (20.1)	75 (13.9)	
Unknown	27 (3.1)	6 (1.8)	21 (3.9)	
**Comorbidities, n (%)**				
Malignancy	72 (8.3)	22 (6.6)	50 (9.3)	0.38
HTN	51 (5.8)	14 (4.2)	37 (6.9)	0.266
Dyslipidemia	40 (4.6)	13 (3.9)	27 (5.0)	0.75
Asthma	25 (2.9)	6 (1.8)	19 (3.5)	0.334
Hypothyroidism	23 (2.6)	9 (2.7)	14 (2.6)	0.996
COPD	22 (2.5)	3 (0.9)	19 (3.5)	0.056
Other comorbidities*	59 (6.8)	17 (5.1)	42 (7.8)	0.163
**Any substance use, n (%)**	314 (36.0)	125 (37.5)	189 (35.1)	0.505
Intravenous drugs, n (%)	13 (1.5)	6 (1.8)	7 (1.3)	0.837
Inhaled drugs, n (%)	167 (19.2)	58 (17.4)	109 (20.2)	0.593
Active smoking, n (%)	226 (25.9)	86 (25.8)	140 (26.0)	0.999
**Coinfections, n (%)**				
Chronic hepatitis B	32 (3.7)	11 (3.3)	21 (3.9)	0.903
Inactive hepatitis B carrier	36 (4.1)	11 (3.3)	25 (4.6)	0.629
Hepatitis C	39 (4.5)	4 (1.2)	35 (6.5)	**0.001**
None	772 (88.5)	308 (92.5)	464 (86.1)	**0.016**
**HIV status and treatment**				
New HIV diagnosis, n (%)	129 (14.8)	69 (21.2)	60 (11.0)	**0.001**
Time since diagnosis, years (IQR)	1.5 (0.1–6.9)	0.5 (0.0–3.8)	3.0 (0.4–8.0)	**<0.001**
**Immunological status at diagnosis**				
CD4 cell count at diagnosis, Median (IQR) (n = 564)	115.0 (47.8–253.0)	57.0 (30.0–129.0)	192.0 (81.2–356.2)	**<0.001**
Stage of infection at diagnosis^a^, n (%)				**<0.001**
Stage I	57 (6.5)	2 (0.6)	55 (10.2)	
Stage II	131 (15.0)	20 (6.0)	111 (20.6)	
Stage III	445 (51.0)	241 (72.4)	204 (37.8)	
Unknown	239 (27.4)	70 (21.0)	169 (31.4)	
**Immunovirological status at admission**				
CD4 cell count, Median (IQR) (n = 723)	192.0 (57.5–378.0)	58.0 (23.0–132.0)	290.0 (146.8–456.0)	**<0.001**
Stage of infection^a^, n (%)				**<0.001**
Stage I	107 (12.3)	0 (0.0)	107 (19.9)	
Stage II	222 (25.5)	0 (0.0)	222 (41.2)	
Stage III	529 (60.7)	333 (100)	196 (36.4)	
Unknown	14 (1.6)	0 (0.0)	14 (2.6)	
CD4 Nadir cell count, Median (IQR) (n = 611)	88.0 (30.0–212.0)	40.0 (17.0–94.0)	156.0 (57.5–323.5)	**<0.001**
HIV viral load, n (%)				**<0.001**
Detectable	478 (54.8)	251 (75.4)	227 (42.1)	
Undetectable (< 50 copies/mL)	287 (32.9)	43 (12.9)	244 (45.3)	
Unknown	107 (12.3)	39 (11.7)	68 (12.6)	
HIV viral load, copies/mL, Median (IQR) (n = 478)	87992 (8397–394 998)	179,000 (38,266–500,876)	41,043 (1,024–210,327)	
**History of ART use, n (%)**	599 (68.7)	178 (53.5)	421 (78.1)	**<0.001**
ART regimen (among treated), n (%) (n = 599)				
INSTI + 2 NRTI	196 (32.7)	59 (34.7)	137 (31.9)	0.579
PI + 2 NRTI	196 (32.7)	47 (27.6)	149 (34.7)	0.117
NNRTI + 2 NRTI	159 (26.5)	47 (27.6)	112 (26.1)	0.778
INSTI + PI	15 (2.5)	4 (2.4)	11 (2.6)	1.000
INSTI + PI + 2 NRTI	6 (1.0)	2 (1.2)	4 (0.9)	0.679
Other/unknown ^b^	27 (4.6)	11 (6.5)	16 (3.7)	0.215
Time on ART, years (IQR) (n = 465)	2.3 (0.6–5.9)	1.4 (0.3–4.8)	2.7 (0.7–6.1)	0.189
**Adherence (among treated), n (%)**				**<0.001**
Suboptimal**	178 (29.7)	89 (50.0)	89 (21.1)	
**Prophylaxis, n (%)**				
*P. jirovecii* prophylaxis	252 (28.9)	130 (39.0)	122 (22.6)	**<0.001**
MAC prophylaxis	48 (5.5)	27 (8.1)	21 (3.9)	**0.03**
**Hospitalization**				
Period of hospitalization, n (%)				**0.036**
**Pre-COVID-19 †**	291 (33.4)	130 (39.0)	161 (29.9)	
**During COVID-19** ‡	417 (47.8)	154 (46.2)	263 (48.8)	
**Post-COVID-19** ‡‡	164 (18.8)	49 (14.7)	115 (21.3)	
Hospital stays, days – median (IQR)	8.0 (4.0–15.0)	14.0 (8.0–25.0)	6.0 (3.0–10.0)	**<0.001**
ICU admission, n (%)	59 (6.8)	38 (11.4)	21 (3.9)	**<0.001**
ICU stay, days Median (IQR) (n = 59)	6.0 (3.0–11.0)	5.5 (3.0–8.8)	7.0 (5.0–13.0)	0.198
Readmission within 30 days, n (%)	111 (12.7)	63 (18.9)	48 (8.9)	**<0.001**
In-hospital Mortality, n (%)	37 (4.2)	27 (8.1)	10 (1.9)	**<0.001**
30-day Mortality, n (%)	42 (4.8)	28 (8.6)	14 (2.6)	**<0.001**

Abbreviations: **ART**, antiretroviral therapy; **COPD**, chronic obstructive pulmonary disease; **HTN**, hypertension; **ICU**, intensive care unit; **INSTI**, integrase strand transfer inhibitor; **IQR**, interquartile range; **MAC**, *Mycobacterium avium* complex; **NNRTI**, non-nucleoside reverse transcriptase inhibitor; **NRTI**, nucleoside/nucleotide reverse transcriptase inhibitor; **PI**, protease inhibitor; **T2DM**, type 2 diabetes mellitus.**Symbols:** *Includes kidney failure, heart failure, T2DM, and obesity. **Suboptimal adherence: less than 95% of doses taken. †Pre-COVID-19: admissions before March 1, 2020. ‡During COVID-19: admissions from March 1, 2020, to May 5, 2023 (date when the WHO declared the end of the global health emergency). ‡‡Post-COVID-19: admissions after May 5, 2023. ^a^Defined according to CDC classification. ^b^Includes atypical combinations, simplification regimens, multidrug rescue regimens, and cases without detailed medication records. Missing/not documented data: sexual orientation, 73/872 (8.4%); educational level, 27/872 (3.1%); stage at HIV diagnosis, 239/872 (27.4%); CD4 count at diagnosis, 308/872 (35.3%); CD4 count at admission, 149/872 (17.1%); CD4 nadir, 261/872 (29.9%); and HIV viral load status at admission, 107/872 (12.3%). Among episodes involving patients receiving ART (n = 599), time on ART was unavailable in 134 (22.4%) and adherence was not documented in 2 (0.3%). Unknown/not documented values were retained as separate categories in descriptive analyses when applicable. Substance-use categories were non-mutually exclusive.

Most episodes occurred in young adults, with a median age of 34 years (IQR, 28–47). Overall, 62.5% of episodes involved individuals aged 18–40 years, and there was a marked male predominance (89.1%; n = 777). Regarding educational attainment, 80.5% of episodes involved individuals who had completed at least secondary education (39.6% secondary, 20.0% technical education, and 19.6% a university/professional degree).

The most frequent comorbidities were malignancy (8.3%; n = 72), hypertension (5.8%; n = 51), and dyslipidemia (4.6%; n = 40). Psychoactive substance use was reported in 36.0% of episodes, most commonly tobacco smoking (25.9%) and inhalant use (19.2%). Regarding coinfections, hepatitis B virus infection was documented in 7.8% of episodes (4.1% inactive carriers and 3.7% chronic infection), and hepatitis C virus infection in 4.5%.

### Immunovirological status and antiretroviral therapy

Among episodes in individuals with a prior HIV diagnosis (85.2%), the median time from HIV diagnosis to hospital admission was 1.5 years (IQR, 0.1–6.9). New HIV diagnoses accounted for 14.8% of episodes (n = 129).

At admission, immune compromise was common. The median CD4 + T-cell count was 192 cells/µL (IQR, 57.5–378), and 60.7% of hospitalization episodes were classified as CDC stage 3 (n = 529) (Table 1).

Overall, 68.7% of episodes (n = 599) involved individuals with prior antiretroviral therapy (ART) use, with a median treatment duration of 2.3 years (IQR, 0.6–5.9). INSTI-based and PI-based regimens were the most common, each accounting for 32.7% (n = 196) of treated episodes. Among episodes with prior ART use, suboptimal adherence was documented in 29.7% (n = 178). Within this subgroup, 52.8% (n = 94) had discontinued ART for >1 month, representing 15.7% of all episodes with prior ART use.

### Causes of hospitalization

Across the study period, non–AIDS-defining conditions accounted for 539 episodes (61.8%), whereas AIDS-defining conditions accounted for 333 episodes (38.2%) (Table 1).

Compared with AIDS-defining episodes, non–AIDS-defining episodes were associated with a more favorable sociodemographic and clinical profile, including: (i) higher educational attainment (technical/university education: 45.5% vs 29.5%); (ii) a more favorable ART history (prior ART use: 78.1% vs 53.4%; optimal adherence: 78.8% vs 50.0%); (iii) longer time since HIV diagnosis (median, 3.0 vs 0.5 years); and (iv) better immunovirological status, including a less severe CD4 nadir (median, 156 vs 40 cells/µL), higher CD4 count at admission (median, 290 vs 58 cells/µL), and better virological control (detectable viral load: 42.1% vs 75.4%).

In contrast, AIDS-defining episodes were associated with worse hospital outcomes, including longer length of stay (median, 14.0 vs 6.0 days), more frequent ICU admission (11.4% vs 3.9%), and higher 30-day mortality (8.6% vs 2.6%) than non–AIDS-defining episodes.

### Temporal trends and the effect of the pandemic

The 872 hospitalization episodes were distributed across the three COVID-19 epidemiological periods as follows: 291 in the pre-pandemic period, 417 during the pandemic, and 164 in the post-pandemic period.

Patient characteristics and outcomes across the study periods are presented in Table 2 (see S2 Table for full details). The proportion of hospitalizations due to AIDS-defining conditions decreased progressively, falling from 44.7% in the pre-pandemic period to 29.9% in the post-pandemic period, a trend compensated by a reciprocal increase in non–AIDS-defining causes (p = 0.006) (Fig 1).

**Table 2:** Baseline characteristics and outcomes of people living with HIV hospitalized at HUSI, Bogotá, Colombia (2018–2024), stratified by COVID-19 pandemic period (n = 872).

Characteristics	Pre-COVID-19 pandemic	During COVID-19 pandemic	Post-COVID-19 pandemic
	Total	Total	Total
	N= 291	N=417	N=164
Male, n (%)	262 (90.0)	370 (88.7)	145 (88.4)
Age, median (IQR)	34.0 (27.0 - 49.0)	34.0 (28.0 - 46.0)	36.0 (29.0 - 49.0)
**Sexual orientation, n (%)**			
Homosexual	140 (48.1)	219 (52.5)	90 (54.9)
Heterosexual	109 (37.5)	132 (31.7)	50 (30.5)
Bisexual	19 (6.5)	25 (6.0)	15 (9.1)
Unknown	23 (7.9)	41 (9.8)	9 (5.5)
**Education level, n (%)**			
University	51 (17.5)	89 (21.3)	31 (18.9)
Technical	60 (20.6)	89 (21.3)	25 (15.2)
High school	120 (41.2)	172 (41.2)	65 (39.6)
Primary education	52 (17.9)	53 (12.7)	37 (22.6)
Unknown	8 (2.7)	14 (3.4)	5 (3.0)
**Comorbidities, n (%)**			
Malignancy	27 (9.3)	29 (7.0)	16 (9.8)
HTN	16 (5.5)	23 (5.5)	12 (7.3)
Dyslipidemia	16 (5.5)	16 (3.8)	8 (4.9)
Asthma	6 (2.1)	14 (3.4)	5 (3.0)
Hypothyroidism	6 (2.1)	12 (2.9)	2 (1.2)
COPD	5 (1.7)	11 (2.6)	0 (0.0)
Other Comorbidities*	21 (7.2)	30 (7.2)	8 (4.9)
**Any substance use, n (%)**			
Intravenous drugs	5 (1.7)	5 (1.2)	3 (1.8)
Inhaled drugs	56 (19.2)	72 (17.3)	39 (23.8)
Active smoking	68 (23.4)	110 (26.4)	48 (29.3)
**Coinfections, n (%)**			
Chronic Hepatitis B	13 (4.5)	14 (3.4)	5 (3.0)
Inactive hepatitis B carrier	15 (5.2)	18 (4.3)	3 (1.8)
Hepatitis C	10 (3.4)	17 (4.1)	12 (7.3)
None	256 (88.0)	369 (88.5)	147 (89.6)
**HIV status and treatment**			
New HIV diagnosis, n (%)	43 (14.8)	65 (15.6)	21 (12.8)
Time since diagnosis, years (IQR)	1.3 (0.2 - 5.8)	1.1 (0.1 - 6.8)	3.8 (0.6 - 9.2)
**Immunological status at diagnosis**			
CD4 cell count at diagnosis, Median (IQR) (n=564)	108.0 (52.0 - 258.0)	110.0 (43.8 - 243.2)	129.0 (49.0 - 256.0)
**Stage of infection at diagnosis, n (%)^a^**			
Stage I	17 (5.8)	29 (7.0)	11 (6.7)
Stage II	52 (17.9)	58 (13.9)	21 (12.8)
Stage III	160 (55.0)	220 (52.8)	65 (39.6)
Unknown	62 (21.3)	110 (26.4)	67 (40.9)
**Immunovirological status at admission**			
CD4 cell count, Median (IQR) (n=723)	152.0 (53.8 - 342.0)	207.0 (75.2 - 391.0)	216.0 (54.0 - 397.0)
Stage of infection, n (%)^a^			
Stage I	27 (9.3)	62 (14.9)	18 (11.0)
Stage II	66 (22.7)	108 (25.9)	48 (29.3)
Stage III	192 (66.0)	243 (58.3)	94 (57.3)
Unknown	6 (2.1)	4 (1.0)	4 (2.4)
CD4 Nadir cell count, Median (IQR) (n=611)	73.0 (29.5 - 193.0)	94.5 (37.2 - 221.8)	89.5 (16.0 - 197.5)
HIV viral load, n (%)			
Detectable	161 (55.3)	230 (55.2)	87 (53.0)
Undetectable (< 50 copies/mL)	91 (31.3)	139 (33.3)	57 (34.8)
Unknown	39 (13.4)	48 (11.5)	20 (12.2)
HIV viral load, copies/mL, Median (IQR) (n=478)	92,045 (4,157 - 432,000)	91,264 (8,123 - 388,521)	85,400 (15,246 - 261,150)
**History of ART use, n (%)**	207 (71.1)	271 (65.0)	121 (73.8)
Time on ART, years (IQR) (n=465)	1.3 (0.4 - 4.6)	2.2 (0.5 - 5.8)	4.1 (2.2 - 8.0)
**ART Regimen (among treated, n)**	**n=207**	**n=271**	**n= 121**
PI + 2 NRTI:	73 (35.3)	91 (33.6)	32 (26.4)
INSTI + 2 NRTI	60 (29.0)	85 (31.4)	51 (42.1)
NNRTI + 2 NRTI	52 (25.1)	85 (31.4)	22 (18.2)
INSTI + PI	7 (3.4)	4 (1.5)	4 (3.3)
INSTI + PI + 2 NRTI:	3 (1.4)	2 (0.7)	1 (0.8)
Other/unknown^b^	12 (5.8)	4 (1.5)	11 (9.1)
**Adherence (among treated), n (%)**	**n=207**	**n=271**	**n= 121**
Suboptimal**	63 (30.4)	70 (25.8)	45 (37.2%)
			
**Prophylaxis, n (%)**			
*P. jirovecii* prophylaxis	101 (34.7)	118 (28.3)	33 (20.1)
MAC prophylaxis	31 (10.7)	15 (3.6)	2 (1.2)
**Hospitalization**			
Hospital stay, days – median (IQR)	8.0 (4.0 - 16.0)	8.0 (4.0 - 15.0)	8.0 (3.8 - 15.0)
ICU admission, n (%)	19 (6.5)	23 (5.5)	17 (10.4)
ICU stay, days Median (IQR)	4.0 (2.5 - 9.5)	9.0 (6.5 - 13.0)	4.0 (3.0 - 6.0)
Readmission within 30 days, n (%)	39 (13.4)	55 (13.2)	17 (10.4)
In-hospital Mortality, n (%)	13 (4.5)	15 (3.6)	9 (5.5)

Abbreviations: **ART:** Antiretroviral therapy; **COPD:** Chronic Obstructive Pulmonary Disease; **HTN:** Hypertension; **ICU:** Intensive Care Unit; **INSTI:** Integrase strand transfer inhibitor; **IQR:** Interquartile range; **MAC:** *Mycobacterium avium* complex; **NNRTI:** Non-nucleoside reverse transcriptase inhibitor; **NRTI:** Nucleoside/nucleotide reverse transcriptase inhibitor; **PI:** Protease inhibitor; **T2DM:** Type 2 Diabetes Mellitus. Symbols: * Other Comorbidities: Includes Kidney failure, Heart failure, T2DM, Obesity. **Suboptimal adherence: Less than 95% of doses taken. **†Pre-COVID-19:** Admissions before March 1, 2020. **‡During COVID-19:** Admissions from March 1, 2020, to May 5, 2023 (date when WHO declared the end of the global health emergency). **‡‡Post-COVID-19:** Admissions after May 5, 2023. ^**a**^Defined according to CDC classification. ^**b**^Includes atypical combinations, simplification regimens, multidrug rescue regimens, and cases without detailed medication records. Missingness for study variables is reported in Table 1; analyses were based on available cases.

**Fig 1:**
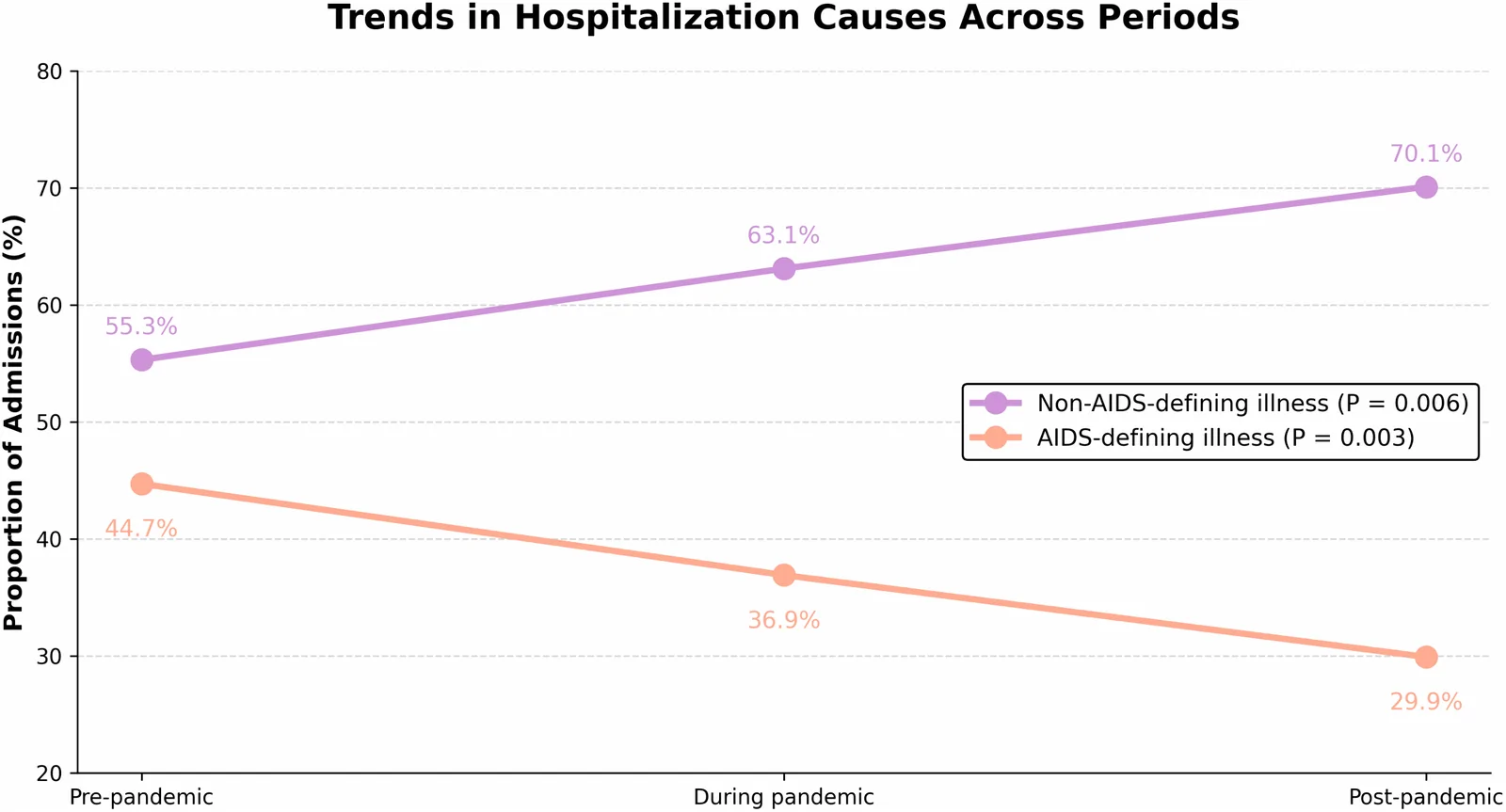
Temporal trends in causes of hospitalization: AIDS-defining vs. non-AIDS-defining events across the COVID-19 pandemic periods.

Changes in patient characteristics across periods When examining the evolution of patient characteristics, notable shifts in behavioral risk factors and treatment patterns were identified.

Regarding behavioral factors, a progressive increase in substance use and coinfections was documented. Active smoking rose from 23.5% (n = 68) in the pre-pandemic period to 29.3% (n = 48) in the post-pandemic period, while inhaled drug use increased from 19.2% to 23.8%. Similarly, the prevalence of hepatitis C virus infection doubled, rising from 3.4% (n = 10) to 7.3% (n = 12) in the final period.

With respect to HIV-related characteristics, the proportion of *de novo* HIV diagnoses showed a slight decrease in the post-pandemic era. When contrasted with the observed increase in time since diagnosis among hospitalized patients, this finding suggests a profile of greater chronicity within the study population. Regarding antiretroviral therapy, the use of integrase strand transfer inhibitor (INSTI)–based regimens increased markedly, significantly displacing protease inhibitor– and non-nucleoside reverse transcriptase inhibitor–based regimens. Despite this therapeutic modernization, rates of suboptimal adherence and presentation with advanced disease remained high and constant across all three periods.

In the subgroup of episodes due to AIDS-defining conditions, the post-pandemic period was characterized by indicators of greater social vulnerability and clinical severity. On one hand, the proportion of patients with only primary education remained high, with an upward trend (30.6%; n = 15; S2 Table); on the other, a higher proportion of ICU admissions and in-hospital mortality was recorded compared to previous periods.

Regarding the distribution of the main causes of admission, although non–AIDS-defining illnesses constituted the largest overall category, AIDS-defining conditions were the leading specific group, accounting for 333 of the 872 episodes (38.2%), followed by non–AIDS-defining infections (n = 293; 33.6%). Gastrointestinal or hepatic conditions (n = 44; 5.0%), neurological disorders (n = 42; 4.8%), surgical conditions (n = 33; 3.8%), and mental health disorders (n = 28; 3.2%) were documented less frequently (S1 Fig)

Among the defining causes of AIDS, tuberculosis (pulmonary and extrapulmonary) was the most frequent etiology (15.8% of the group), closely followed by *Pneumocystis jirovecii* pneumonia (14.9%), cytomegalovirus disease (13.8%), and Kaposi sarcoma (10.6%) (Table 3).

**Table 3:** Primary causes of admission of people living with HIV hospitalized at HUSI, Bogotá, Colombia (2018–2024), stratified by COVID-19 pandemic period (n = 872).

Cause of admission	Total (n=872)	Pre-COVID-19 pandemic n=291 (33.4%)	During COVID-19 pandemic n=417 (47.8%)	Post-COVID-19 pandemic n=164 (18.8%)	P value
**AIDS-defining illness, n (%)**	333 (38.2)	130 (44.7)	154 (36.9)	49 (29.9)	**0.003**
**Type of AIDS-defining illness, n (%)**					
Tuberculosis (any site)	55 (15.8)	17 (12.5)	23 (14.6)	15 (27.3)	
*Pneumocystis jirovecii* pneumonia	52 (14.9)	23 (16.9)	21 (13.3)	8 (14.5)	
Cytomegalovirus disease	48 (13.8)	18 (13.2)	27 (17.1)	3 (5.5)	
Kaposi sarcoma	37 (10.6)	16 (11.8)	12 (7.6)	9 (16.4)	
Cerebral toxoplasmosis	36 (10.3)	17 (12.5)	16 (10.1)	3 (5.5)	
Disseminated cryptococcosis	23 (6.6)	10 (7.4)	7 (4.4)	6 (10.9)	
Chronic intestinal Cryptosporidiosis	17 (4.9)	3 (2.2)	11 (7.0)	3 (5.5)	
HIV wasting syndrome	17 (4.9)	3 (2.2)	11 (7.0)	3 (5.5)	
Disseminated histoplasmosis	17 (4.9)	8 (5.9)	8 (5.1)	1 (1.8)	
Esophageal candidiasis	16 (4.6)	10 (7.4)	5 (3.2)	1 (1.8)	
Non-Hodgkin lymphoma	15 (4.3)	4 (2.9)	9 (5.7)	2 (3.6)	
Primary CNS lymphoma	5 (1.4)	4 (2.9)	1 (0.6)	0 (0.0)	
Recurrent pneumonia	3 (0.9)	1 (0.7)	2 (1.3)	0 (0.0)	
Salmonella septicemia	3 (0.9)	1 (0.7)	2 (1.3)	0 (0.0)	
HIV-related encephalopathy	2 (0.6)	0 (0.0)	2 (1.3)	0 (0.0)	
Mycobacterium (other species)	2 (0.6)	1 (0.7)	0 (0.0)	1 (1.8)	
**Non-AIDS defining illness, n (%)**	539 (61.8)	161 (55.3)	263 (63.1)	115 (70.1)	**0.006**
**Type of non-AIDS-defining illness, n (%)**					
Non-AIDS defining infection	293 (54.4)	90 (55.9)	143 (54.4)	60 (52.2%)	
Gastrointestinal causes	44 (8.2)	19 (11.8)	18 (6.8)	7 (6.1%)	
Neurological causes	42 (7.8)	12 (7.5)	23 (8.7)	7 (6.1%)	
Surgical conditions	33 (6.1)	7 (4.3)	22 (8.4)	4 (3.5%)	
Mental health	28 (5.2)	7 (4.3)	12 (4.6)	9 (7.8%)	
Non-HIV-related neoplasms	19 (3.5)	4 (2.5)	11 (4.2)	4 (3.5%)	
Pulmonary causes	19 (3.5)	5 (3.1)	9 (3.4)	5 (4.3%)	
Cardiovascular causes	9 (1.7)	4 (2.5)	3 (1.1)	2 (1.7%)	
Adverse effects of therapy	9 (1.7)	2 (1.2)	4 (1.5)	3 (2.6%)	
Musculoskeletal injuries	8 (1.5)	2 (1.2)	5 (1.9)	1 (0.9%)	
Other causes*	64 (11.9)	20 (12.4)	26 (9.9)	18 (15.7)	

Abbreviations: **AKI**, acute kidney injury; **CNS**, central nervous system; **MAC**, *Mycobacterium avium* complex; **PML**, progressive multifocal leukoencephalopathy. **Note:** More than one principal diagnosis could be assigned to a single admission when two conditions contributed equally to the reason for hospitalization; therefore, the sum of the individual categories may exceed the total number of admissions. Percentages for non-AIDS-defining illnesses were calculated based on the total number of non-AIDS-defining diagnoses. *Includes genitourinary causes, renal disease, autoimmune conditions, metabolic/endocrine diseases, poisoning/external causes, and other miscellaneous conditions.

Regarding changes in AIDS-defining conditions over the study period, we observed a clear shift in their distribution. Tuberculosis (all forms) increased substantially, from 12.5% in the pre-pandemic period to 27.3% in the post-pandemic period, becoming the leading cause in the latter. In contrast, *Pneumocystis jirovecii* pneumonia, which was the most frequent AIDS-defining condition in the pre-pandemic period (16.9%), decreased to third place in the post-pandemic period (14.5%). Kaposi sarcoma showed an upward trend, increasing from 11.8% to 16.4%, and ranked as the second most frequent AIDS-defining condition in the post-pandemic period (Fig 2).

**Fig 2:**
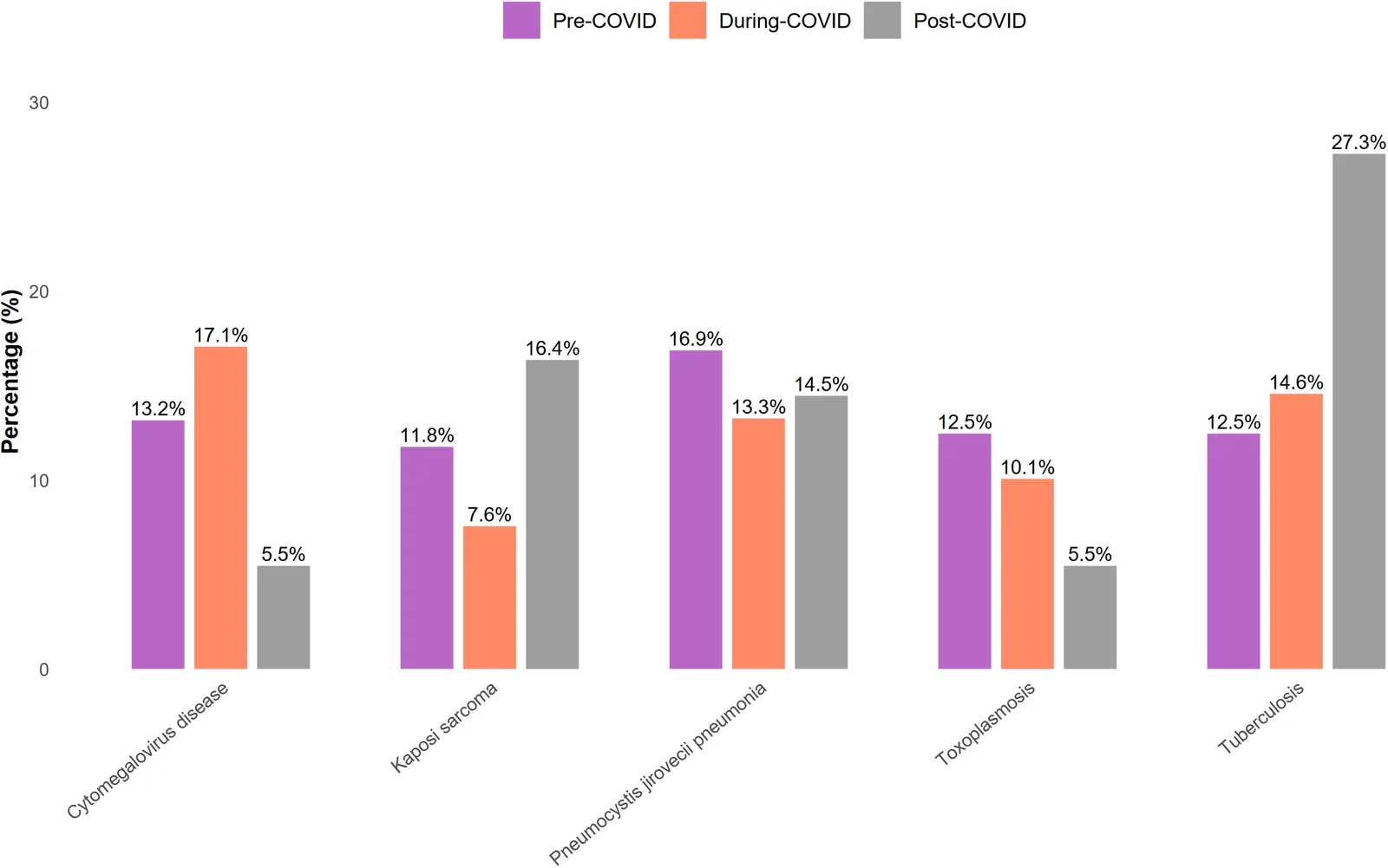
Top 5 AIDS-defining causes of admission.

Among the 539 episodes attributed to non–AIDS-defining conditions, non-opportunistic infections were the predominant category, accounting for 54.4% (n = 293). Within this category, gastrointestinal infections were the most frequent (27.0%; n = 79), followed by pneumonia (15.7%; n = 46) (S2 Fig).

Although the overall mix of non–AIDS-defining causes was largely stable across periods, admissions for mental health disorders increased in the post-pandemic period (5.5%) compared with the pre-pandemic period (2.4%). This increase coincided with a downward trend in non-opportunistic infections (Fig 3).

**Fig 3:**
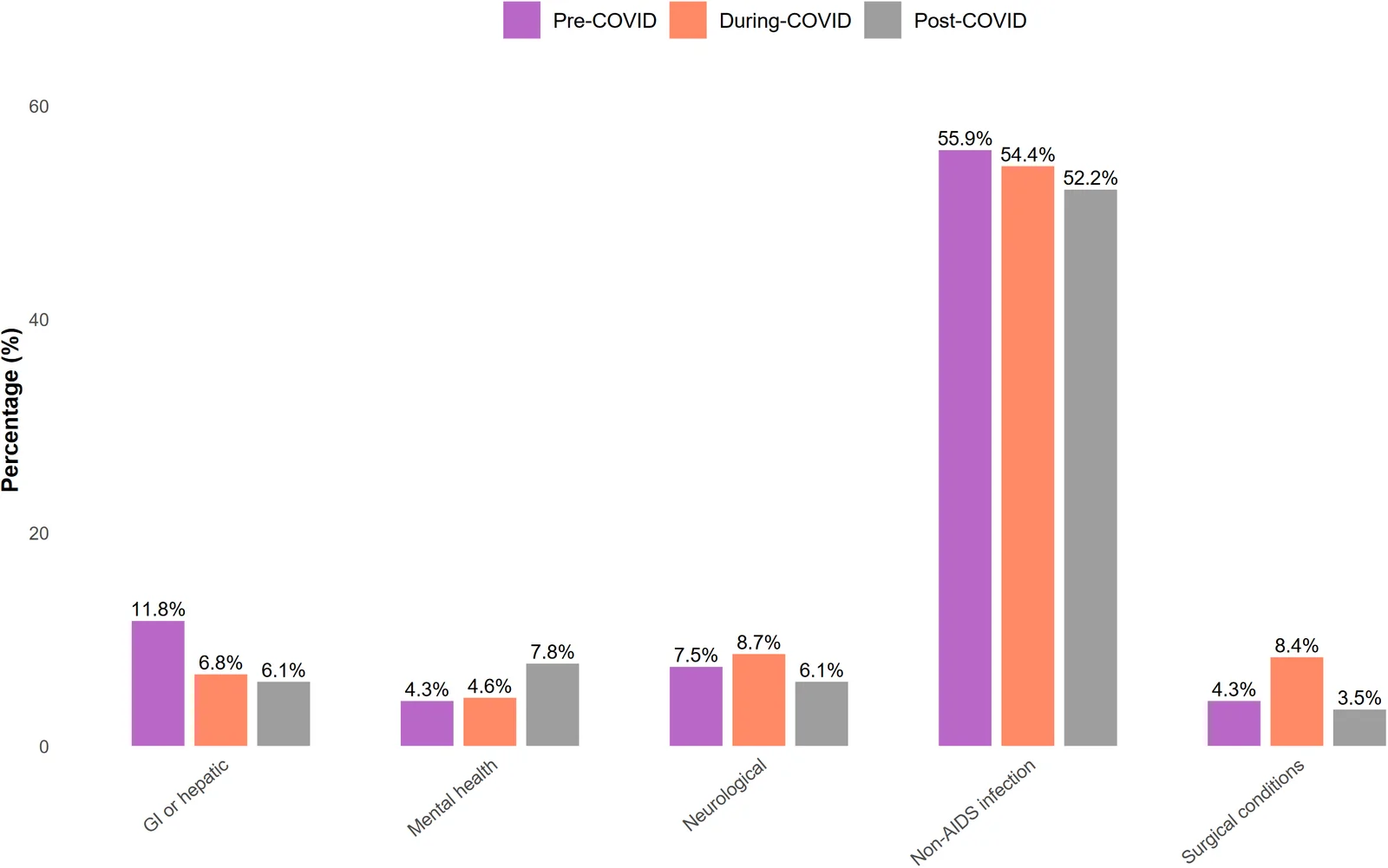
Top 5 non-AIDS-defining causes of admission.

In addition to the primary cause of admission, secondary concomitant conditions were identified in 23.7% of episodes. The most frequent were non–AIDS-defining infections (n = 66), wasting syndrome (n = 41), and esophageal candidiasis (n = 17) (S1 Table).

### Hospital outcomes

The median length of hospital stay was 8.0 days (IQR, 4.0–15.0). Overall, 6.8% of episodes (n = 59) required admission to the intensive care unit (ICU), with a median ICU stay of 6.0 days (IQR, 3.0–11.0). Readmission to the same institution within 30 days of discharge occurred in 111 episodes (12.7%). Of these, 71 (64.0%) were related to the same cause as the index hospitalization, whereas 40 (36.0%) were attributed to different primary causes. In-hospital mortality was 4.2% (n = 37), and 30-day mortality was 4.8% (n = 42). Thirty-day mortality was significantly higher among episodes due to AIDS-defining conditions (8.6% vs. 2.6%; p < 0.001). Across periods, in-hospital mortality remained stable: 13 events (4.5%) in the pre-pandemic period, 15 (3.6%) during the pandemic, and 9 (5.5%) in the post-pandemic period.

## Discussion

In this cohort of hospitalizations at a tertiary-care referral center, non–AIDS-defining conditions accounted for the majority of admissions (61.8%) and became progressively more frequent across the periods evaluated. Although the overall frequency of AIDS-defining conditions decreased, these conditions remained associated with greater clinical severity, as reflected by longer hospital stays and higher 30-day mortality (8.6% vs. 2.6%).

This morbidity profile suggests a local evolution consistent with prior national reports. Whereas Montúfar-Andrade et al. (2009–2011) and Álvarez et al. (2014–2015) reported AIDS-defining admissions in 66% and 54.6% of cases, respectively [10,24], our cohort documented a lower overall proportion (38.2%), with a decline within our center from 44.7% in the pre-pandemic period to 29.9% in the post-pandemic period.

However, this transition has not been uniform across Latin America. Meta-analyses published before 2015 estimated that AIDS-defining conditions accounted for approximately 40% of admissions (range, 15%–65%) [25], whereas more recent pooled estimates have reported proportions as high as 62.4% (52.6%–71.3%) [8], likely reflecting heterogeneity across health systems. These differences are evident at the cohort level. In Rio de Janeiro, Coelho et al. (2007–2013) described a pattern similar to ours, with a reduction in AIDS-defining admissions from 49.2% to 38.3% and a parallel rise in non–AIDS-defining admissions [26]. In contrast, cohorts in Mexico and Brazil have continued to report proportions above 50%, contributing to the marked variability in regional aggregate estimates [27,28].

Overall, our findings suggest that this hospital reflects an ongoing epidemiological transition. The proportion of AIDS-defining admissions is lower than historical regional estimates (≈62%) [8] and aligns with reports from other Latin American academic referral centers (Brazil) [26] and recent evidence from Ecuador (37.3%), even when considering methodological differences [29]. Nonetheless, these proportions remain substantially higher than the < 15%–30% reported in North America and Western Europe [7,8,25,30,31], underscoring the need to strengthen early diagnosis strategies and retention in care to close the remaining gap.

This positive trend may be partly explained by improvements in the HIV care cascade in Colombia. During 2014–2015, although ART coverage exceeded 80%, viral suppression was achieved in only 50%–55% of cases [32,33]. By 2023–2024, 80.6% of people receiving ART achieved viral suppression [9]. Despite these gains, our results highlight a gap between population indicators and the hospitalized population: although 68.7% of episodes involved individuals with prior ART use, more than half presented with detectable viral loads, particularly those admitted with AIDS-defining conditions. This pattern suggests persistent challenges in adherence, access, or continuity of ART among individuals who ultimately require hospitalization, limiting a full transition toward the chronic comorbidity pattern observed in high-income settings. Late diagnosis likely contributes as well, as reported regionally by Belaunzarán-Zamudio et al., and is consistent with our cohort, in which 51% of patients had an advanced clinical stage at the time of diagnosis [34].

Within AIDS-defining conditions, tuberculosis remains the most frequent etiology globally (19%), followed by bacterial pneumonia and *Pneumocystis jirovecii* infection [8]. This distribution closely mirrors our findings, where tuberculosis (15.8%) and *P. jirovecii* pneumonia (14.9%) ranked first and second, supporting the persistence of advanced disease despite expanded ART coverage. In Colombia, tuberculosis is the leading cause of hospitalization (23.3%–37%) and mortality in most series [10,24,35], although regional heterogeneity exists. Two local studies reported cerebral toxoplasmosis as the leading cause of admission, diverging from the broader Latin American pattern in which tuberculosis predominates [36,37].

We examined causes of hospitalization across pre-pandemic, pandemic, and post-pandemic periods. Although some patterns likely reflect secular trends, the increase in tuberculosis admissions is notable and consistent with the rebound in tuberculosis detection and reporting following the COVID-19–related disruptions in health services described globally and regionally [38]. The burden of tuberculosis/HIV coinfection is substantial; globally, it is estimated that approximately one-quarter of people with HIV may have active tuberculosis [39]. Locally, Puentes-Ariza et al. reported that 52.3% of tuberculosis cases in a high-complexity hospital in Bogotá occurred among PLWH [40], highlighting the clinical and programmatic synergy between these conditions and the need for integrated screening strategies.

Improved detection is associated with updated national guidelines prioritizing molecular testing [41] and the pandemic-driven expansion of laboratory capacity. These techniques increase sensitivity by 23% in paucibacillary disease—common in HIV—contributing to the higher number of identified cases [42].

Regarding AIDS-defining malignancies, Kaposi sarcoma increased steadily in our cohort, reaching 16.4% in the post-pandemic period, exceeding the global average reported across studies (10.6%). This contrasts with historical local trends and with high-income settings, where ART has been associated with an approximately 70% reduction in the incidence of AIDS-defining cancers [43] and where Colombian series previously reported Kaposi sarcoma as the cause of admission in only 2.0%–4.0% of cases [10,24,44]. Nonetheless, this burden is consistent with the Latin American pattern described by the CCASAnet network, in which Kaposi sarcoma remains the most frequent HIV-associated neoplasm and accounts for 55% of cancer diagnoses in the region [45]. Recent reports from Peru similarly describe advanced presentations, with visceral involvement in 57.4% of cases, closely linked to lack of prior ART and late diagnosis [46].

Among non–AIDS-defining conditions, non-opportunistic infections were the leading cause of hospitalization, although their relative frequency decreased slightly across periods. This aligns with a recent meta-analysis identifying bacterial infections as the second leading cause of hospitalization globally (26%) [8] and with North American series (2005–2015) reporting approximately 25% of admissions due to bacterial infections [8]. In Latin America, the burden is often higher; for example, in Rio de Janeiro, bacterial infections accounted for 53.4% of admissions, whereas in our cohort they represented 33.6%—still substantial, though lower than that report [26].

The observed increase in mental health–related admissions warrants particular attention, given the bidirectional relationship between psychopathology, substance use, adherence, and outcomes. Colombian evidence (Medellín, 2010–2016) has identified psychiatric disorders, social instability, and substance dependence as key determinants of ART nonadherence [10]. In this context, the rise in psychiatric causes—especially in the post-pandemic period—may indicate an emerging pattern resembling that of high-income settings, where neuropsychiatric morbidity becomes more prominent as populations age and move beyond advanced AIDS.

This pattern is consistent with international evidence. A meta-analysis reported that psychiatric disorders accounted for 13% of hospitalizations in Europe (2007–2015) [25], and in New York, psychiatric services were involved in 15.1% of admissions, ranking as the primary cause in 7.2% [30]. London cohorts have also reported a substantial burden of depression and self-harm, exacerbated by substance use [7]. The concurrent rise in inhalant use and smoking observed in our cohort underscores a public health challenge that requires integrated harm-reduction and psychosocial support strategies beyond virological control.

Despite improvements in outpatient management, mortality among hospitalized people with HIV remains substantial globally. Burke et al. (2014–2023) estimated an overall mortality rate of 17% in adults, with regional variation [8]. In our cohort, 30-day mortality was 4.8%, at the lower end of the range reported nationally [8,10,24], consistent with the availability of tertiary-care resources and potentially effective inpatient management. However, within the subgroup admitted with AIDS-defining conditions, case-fatality increased in the post-pandemic period (from 7.7% to 14.3%). This pattern may reflect delayed care and persistent late diagnosis, although the observational design does not allow causal inference.

Overall, the persistence of AIDS -defining conditions— together with the increase in mortality within this subgroup—highlights ongoing structural gaps in HIV care. Closing the gap with international benchmarks will likely require strategies that extend beyond medication availability, including active case finding, early diagnosis, psychosocial support to improve adherence, and multidisciplinary models of care.

### Limitations

This study has limitations inherent to its retrospective, single-center design. First, reliance on clinical records may introduce information bias. To mitigate this risk, medical records underwent peer review and systematic triangulation of information, integrating outpatient and inpatient documentation as well as assessments by social work and psychology teams, which provided complementary detail on sociodemographic, psychosocial, and clinical variables. ART adherence was based on self-report documented during routine clinical care and was therefore subject to recall and social-desirability bias, potentially resulting in overestimation of adherence. Admission HIV viral load provided an objective complementary marker of treatment response. Substance-use information was also largely self-reported and was not confirmed by toxicological testing; therefore, underreporting or misclassification cannot be excluded.

Second, as a tertiary-care university referral center, HUSI likely concentrates more severe cases than lower levels of care. This may overestimate the frequency and severity of AIDS-defining conditions (e.g., visceral Kaposi sarcoma) and may also influence mortality estimates; therefore, generalizability to less complex hospitals is limited.

Third, the analysis of COVID-19–related changes relied on chronological periods defined by administrative milestones, without the ability to fully control for potential confounders such as insurance changes, service reorganization, migration flows, or ongoing improvements in outpatient care. Accordingly, the role of the pandemic should be interpreted cautiously. Our findings are consistent with a combination of secular improvements in the HIV care cascade and pandemic-related disruptions. Although delayed care during COVID-19 peaks and the broader socioeconomic impact of the pandemic may have contributed to later and more severe presentations in the recovery period, the observational design and incomplete information on all confounders preclude attributing these changes solely to the pandemic.

Fourth, excluding patients transferred to another institution within the first 72 hours may have introduced selection bias. Although this criterion was necessary to ensure complete ascertainment of clinical outcomes, early transfers in our healthcare system are often driven by insurance-related administrative processes rather than clinical severity. This exclusion may therefore have limited representation of the full clinical spectrum and reduced the generalizability of our findings. However, given the relatively small proportion of excluded cases (7%), we expect any resulting effect on our main findings to have been modest.

Despite these limitations, strengths include the cohort size, systematic inclusion of all hospitalization episodes over an extended period, and rigorous clinical classification of admission causes, providing robust local evidence in a setting of epidemiological transition and useful inputs for service planning and public health decision-making.

## Conclusions

Our study documents an ongoing epidemiological transition in causes of hospitalization among people living with HIV in a tertiary-care center in Colombia. Although AIDS-defining conditions—particularly tuberculosis—continue to account for a substantial proportion of admissions, their relative frequency declined markedly in the post-pandemic period. This pattern resembles trends described in high-income settings; however, the persistent burden of tuberculosis, Kaposi sarcoma, and late diagnosis suggests ongoing structural gaps in HIV care in low- and middle-income contexts.

At the same time, non–AIDS-defining admissions increased, including a growing contribution from mental health–related conditions and substance use, particularly in the post-pandemic period. Collectively, these findings support the need for comprehensive care models that strengthen early diagnosis, tuberculosis prevention and active screening, mental health services, harm reduction, and retention in care to reduce preventable hospitalizations and avoidable mortality among people living with HIV.

## Supporting information

S1 Fig Main causes of hospitalization at Hospital Universitario San Ignacio (HUSI), 2018–2024. (TIF)

S2 Fig Frequency and temporal distribution of non–AIDS-defining infections. The analysis is based on 293 hospitalization episodes due to non–AIDS-defining infectious causes. A total of 308 diagnoses were documented, reflecting multiple coinfections in 17 episodes. The y-axis displays the absolute (n) and relative (%) frequency of each infection calculated based on the total number of episodes (N = 293). Bars represent the distribution stratified by epidemiological period.
(TIF)

S3 Fig Study flow diagram of hospitalization episodes among people living with HIV, HUSI, Bogotá, 2018–2024. (TIF)

S1 Table Secondary causes of admission of people living with HIV hospitalized at HUSI, Bogotá, Colombia (2018–2024), stratified by COVID-19 pandemic period (n = 872). Abbreviations: AKI, acute kidney injury; CNS, central nervous system; MAC, *Mycobacterium avium* complex; PML, Progressive multifocal leukoencephalopathy. Note: Because multiple secondary diagnoses could coexist during the same hospitalization, the sum of the individual categories may exceed the total number of patients.
(DOCX)

S2 Table Baseline characteristics and outcomes of people living with HIV hospitalized at HUSI, Bogotá, Colombia (2018–2024), stratified by admission cause and COVID-19 pandemic period. This table serves as a complement to Table 2, presenting the complete detailed data for all sociodemographic and clinical variables across the three epidemiological periods.
(DOCX)

S1 Dataset De-identified dataset containing the demographic and clinical data of the 872 hospitalization episodes analyzed in the study. (XLSX)
