# Supplementary material for: Causes of hospitalization among people living with HIV in a tertiary-care hospital in Bogotá, Colombia, 2018–2024
Source: PLoS One. 2026 Sep 30;21(9):e0357788. doi: 10.1371/journal.pone.0357788 (PMC13626370; doi:10.1371/journal.pone.0357788)
Supplement: Supplementary file 1 [file pone.0357788.s004.docx]

**S1 Table. Secondary causes of admission of people living with HIV hospitalized at HUSI, Bogotá, Colombia (2018–2024), stratified by COVID-19 pandemic period (n = 872).**

| **Cause of admission** | **Pre-COVID 19 pandemic n=291 (33.4%)** | **During-COVID 19 pandemic n=417 (47.8%)** | **Post-COVID 19 pandemic  n=164 (18.8%)** |
| --- | --- | --- | --- |
| **AIDS-related cause, n (%)** |  |  |  |
| Candidiasis of bronchi/trachea/lungs | 0 (0.0) | 0 (0.0) | 0 (0.0) |
| Esophageal candidiasis | 9 (3.1) | 7 (1.7) | 1 (0.6) |
| Cervical cancer, invasive | 0 (0.0) | 0 (0.0) | 0 (0.0) |
| Disseminated/extrapulmonary coccidioidomycosis | 0 (0.0) | 0 (0.0) | 0 (0.0) |
| Extrapulmonary cryptococcosis | 2 (0.7) | 1 (0.2) | 0 (0.0) |
| Chronic intestinal cryptosporidiosis | 2 (0.7) | 3 (0.7) | 0 (0.0) |
| Cytomegalovirus disease | 7 (2.4) | 8 (1.9) | 1 (0.6) |
| Cytomegalovirus retinitis | 1 (0.3) | 0 (0.0) | 0 (0.0) |
| HIV-related encephalopathy | 1 (0.3) | 0 (0.0) | 0 (0.0) |
| Herpes simplex (chronic/visceral) | 2 (0.7) | 0 (0.0) | 0 (0.0) |
| Disseminated/extrapulmonary histoplasmosis | 1 (0.3) | 2 (0.5) | 0 (0.0) |
| Chronic intestinal isosporiasis | 0 (0.0) | 0 (0.0) | 0 (0.0) |
| Kaposi sarcoma | 6 (2.1) | 6 (1.4) | 1 (0.6) |
| Burkitt lymphoma | 0 (0.0) | 1 (0.2) | 0 (0.0) |
| Immunoblastic lymphoma | 1 (0.3) | 0 (0.0) | 0 (0.0) |
| Primary CNS lymphoma | 0 (0.0) | 0 (0.0) | 0 (0.0) |
| Disseminated MAC / M. kansasii | 0 (0.0) | 0 (0.0) | 0 (0.0) |
| Tuberculosis (any site) | 4 (1.4) | 2 (0.5) | 0 (0.0) |
| Mycobacterium (other species) | 0 (0.0) | 0 (0.0) | 0 (0.0) |
| Pneumocystis jirovecii pneumonia | 4 (1.4) | 3 (0.7) | 1 (0.6) |
| Recurrent pneumonia | 0 (0.0) | 0 (0.0) | 0 (0.0) |
| Progressive multifocal leukoencephalopathy | 0 (0.0) | 0 (0.0) | 0 (0.0) |
| Salmonella septicemia, recurrent | 1 (0.3) | 0 (0.0) | 1 (0.6) |
| Toxoplasmosis of brain | 0 (0.0) | 2 (0.5) | 0 (0.0) |
| HIV wasting syndrome | 13 (4.5) | 24 (5.8) | 4 (2.4) |
| **Other HIV related cause, n (%)** |  |  |  |
| HIV-associated nephropathy | 0 (0.0) | 0 (0.0) | 0 (0.0) |
| AKI associated with HIV | 0 (0.0) | 0 (0.0) | 0 (0.0) |
| Adverse effects of ART/HIV medication | 2 (0.7) | 0 (0.0) | 0 (0.0) |
| **ART adverse effect detail, n (%)** |  |  |  |
| Pancreatitis | 0 (0.0) | 0 (0.0) | 0 (0.0) |
| Erythroderma | 0 (0.0) | 0 (0.0) | 0 (0.0) |
| Hepatitis | 0 (0.0) | 0 (0.0) | 0 (0.0) |
| Neuropsychiatric disorders | 0 (0.0) | 0 (0.0) | 0 (0.0) |
| AKI secondary to ART | 2 (0.7) | 0 (0.0) | 0 (0.0) |
| Other | 0 (0.0) | 0 (0.0) | 0 (0.0) |
| **Non-HIV related cause, n (%)** |  |  |  |
| Non-AIDS-defining infection | 21 (7.2) | 35 (8.4) | 10 (6.1) |
| Cardiovascular causes | 1 (0.3) | 2 (0.5) | 0 (0.0) |
| Pulmonary causes | 0 (0.0) | 2 (0.5) | 0 (0.0) |
| Neurological causes | 5 (1.7) | 2 (0.5) | 0 (0.0) |
| GI or Hepatic causes | 6 (2.1) | 4 (1.0) | 2 (1.2) |
| Surgical conditions | 1 (0.3) | 2 (0.5) | 0 (0.0) |
| Poisoning/external causes | 0 (0.0) | 0 (0.0) | 0 (0.0) |
| Non-HIV-related neoplasms | 3 (1.0) | 4 (1.0) | 2 (1.2) |
| Genitourinary causes | 0 (0.0) | 1 (0.2) | 0 (0.0) |
| Mental health | 3 (1.0) | 1 (0.2) | 0 (0.0) |
| Metabolic/endocrine disease | 0 (0.0) | 0 (0.0) | 0 (0.0) |
| Musculoskeletal injuries | 0 (0.0) | 0 (0.0) | 0 (0.0) |
| Adverse effects of therapy | 2 (0.7) | 2 (0.5) | 0 (0.0) |
| Renal disease | 0 (0.0) | 2 (0.5) | 0 (0.0) |
| Autoimmune condition | 0 (0.0) | 0 (0.0) | 0 (0.0) |
| Other | 8 (2.7) | 12 (2.9) | 2 (1.2) |

AKI: acute kidney injury, CNS: central nervous system, MAC: Mycobacterium avium complex, PML: Progressive multifocal leukoencephalopathy. Note: Because multiple secondary diagnoses could coexist during the same hospitalization, the sum of the individual categories may exceed the total number of patients.
