# Supplementary material for: Causes of hospitalization among people living with HIV in a tertiary-care hospital in Bogotá, Colombia, 2018–2024
Source: PLoS One. 2026 Sep 30;21(9):e0357788. doi: 10.1371/journal.pone.0357788 (PMC13626370; doi:10.1371/journal.pone.0357788)
Supplement: Supplementary file 2 [file pone.0357788.s005.docx]

**S2 Table. Baseline characteristics and outcomes of people living with HIV hospitalized at HUSI, Bogotá, Colombia (2018–2024), stratified by admission cause and COVID-19 pandemic period.**

| **Characteristics** | **Pre-COVID-19 pandemic** | | | | | | | | **During COVID 19 pandemic** | | | | | | | | | | | | | | | | | **Post COVID -19 pandemic** | | | | | | |  |  |
| --- | --- | --- | --- | --- | --- | --- | --- | --- | --- | --- | --- | --- | --- | --- | --- | --- | --- | --- | --- | --- | --- | --- | --- | --- | --- | --- | --- | --- | --- | --- | --- | --- | --- | --- |
|  | **Total** | | **AIDS defining illness** | | | **Non-AIDS defining illness** | | | **Total** | | | | | **AIDS defining illness** | | | | | | | | | **Non-AIDS defining illness** | | | **Total (n=164)** | | **AIDS defining illness** | | **Non-AIDS defining illness** | | |  |  |
|  | **N=291** | | **N=130 (44.7%)** | | | **N=161 (55.3%)** | | | **N=417** | | | | | **N=154 (36.9%)** | | | | | | | | | **N=263 (63.1%)** | | |  |  | **N=49 (29.8%)** | | **N=115 (70.1%)** | | |  |  |
| Male, n (%) | 262 (90.0) | | 114 (87.7) | | | 148 (91.9) | | | 370 (88.7) | | | | | 126 (81.8) | | | | | | | | | 244 (92.8) | | | 145 (88.4) | | 44 (89.8) | | 101 (87.8) | | |  |  |
| Age, median (IQR) | 34.0 (27.0 - 49.0) | | 34.0 (28.0–46.0) | | | 33.0 (26.0–49.0) | | | 34.0 (28.0 - 46.0) | | | | | 36.0 (29.2–46.0) | | | | | | | | | 32.0 (27.0–45.0) | | | 36.0 (29.0 - 49.0) | | 35.0 (29.0–45.0) | | 36.0 (29.0–49.5) | | |  |  |
| **Sexual orientation, n (%)** | | | |  | | |  | | | | |  | | | | | | | |  | | | | | | | | | | | | | |  |
| Homosexual | 140 (48.1) | | 54 (41.5) | | | 86 (53.4) | | | 219 (52.5) | | | | | 68 (44.2) | | | | | | | | | 151 (57.4) | | | 90 (54.9) | | 27 (55.1) | | 63 (54.8) | | |  |  |
| Heterosexual | 109 (37.5) | | 60 (46.2) | | | 49 (30.4) | | | 132 (31.7) | | | | | 65 (42.2) | | | | | | | | | 67 (25.5) | | | 50 (30.5) | | 14 (28.6) | | 36 (31.3) | | |  |  |
| Bisexual | 19 (6.5) | | 8 (6.2) | | | 11 (6.8) | | | 25 (6.0) | | | | | 9 (5.8) | | | | | | | | | 16 (6.1) | | | 15 (9.1) | | 6 (12.2) | | 9 (7.8) | | |  |  |
| Unknown | 23 (7.9) | | 8 (6.2) | | | 15 (9.3) | | | 41 (9.8) | | | | | 12 (7.8) | | | | | | | | | 29 (11) | | | 9 (5.5) | | 2 (4.1) | | 7 (6.1) | | |  |  |
| **Education level, n (%)** |  | |  | | |  | | |  | | | | |  | | | | | | | | |  | | |  | |  | |  | | |  |  |
| University | 51 (17.5) | | 17 (13.1) | | | 34 (21.1) | | | 89 (21.3) | | | | | 20 (13.0) | | | | | | | | | 69 (26.2) | | | 31 (18.9) | | 4 (8.2) | | 27 (23.5) | | |  |  |
| Technical | 60 (20.6) | | 25 (19.2) | | | 35 (21.7) | | | 89 (21.3) | | | | | 30 (19.5) | | | | | | | | | 59 (22.4) | | | 25 (15.2) | | 3 (6.1) | | 22 (19.1) | | |  |  |
| High school | 120 (41.2) | | 54 (41.5) | | | 66 (41.0) | | | 172 (41.2 | | | | | 81 (52.6) | | | | | | | | | 91 (34.6) | | | 65 (39.6) | | 26 (53.1) | | 39 (33.9) | | |  |  |
| Primary education | 52 (17.9) | | 31 (23.8) | | | 21 (13.0) | | | 53 (12.7) | | | | | 21 (13.6) | | | | | | | | | 32 (12.2) | | | 37 (22.6) | | 15 (30.6) | | 22 (19.1) | | |  |  |
| Unknown | 8 (2.7) | | 3 (2.3) | | | 5 (3.1) | | | 14 (3.4) | | | | | 2 (1.3) | | | | | | | | | 12 (4.6) | | | 5 (3.0) | | 1 (2.0) | | 4 (3.5) | | |  |  |
| **Comorbidities, n (%)** |  | |  | | |  | | |  | | | | |  | | | | | | | | |  | | |  | |  | |  | | |  |  |
| Malignancy | 27 (9.3) | | 10 (7.7) | | | 17 (10.6) | | | 29 (7.0) | | | | | 9 (5.8) | | | | | | | | | 20 (7.6) | | | 16 (9.8) | | 3 (6.1) | | 13 (11.3) | | |  |  |
| HTN | 16 (5.5) | | 5 (3.8) | | | 11 (6.8) | | | 23 (5.5) | | | | | 8 (5.2) | | | | | | | | | 15 (5.7) | | | 12 (7.3) | | 1 (2.0) | | 11 (9.6) | | |  |  |
| Dyslipidemia | 16 (5.5) | | 9 (6.9) | | | 7 (4.3) | | | 16 (3.8) | | | | | 4 (2.6) | | | | | | | | | 12 (4.6) | | | 8 (4.9) | | 0 (0.0) | | 8 (7.0) | | |  |  |
| Asthma | 6 (2.1) | | 4 (3.1) | | | 5 (3.1) | | | 14 (3.4) | | | | | 2 (1.3) | | | | | | | | | 12 (4.6) | | | 5 (3.0) | | 0 (0.0) | | 2 (1.7) | | |  |  |
| Hypothyroidism | 6 (2.1) | | 1 (0.8) | | | 4 (2.5) | | | 12 (2.9) | | | | | 7 (4.5) | | | | | | | | | 7 (2.7) | | | 2 (1.2) | | 1 (2.0) | | 3 (2.6) | | |  |  |
| COPD | 5 (1.7) | | 1 (0.8) | | | 5 (3.1) | | | 11 (2.6) | | | | | 2 (1.3) | | | | | | | | | 9 (3.4) | | | 0 (0.0) | | 0 (0.0) | | 5 (4.3) | | |  |  |
| Kidney failure | 2 (0.7) | | 4 (3.1) | | | 5 (3.1) | | | 6 (1.4) | | | | | 1 (0.6) | | | | | | | | | 9 (3.4) | | | 0 (0.0) | | 0 (0.0) | | 2 (1.7) | | |  |  |
| Heart failure | 1 (0.3) | | 3 (2.3) | | | 7 (4.3) | | | 6 (1.4) | | | | | 2 (1.3) | | | | | | | | | 6 (2.3) | | | 1 (0.6) | | 0 (0.0) | | 3 (2.6) | | |  |  |
| T2DM | 4 (1.4) | | 1 (0.8) | | | 4 (2.5) | | | 1 (0.2) | | | | | 5 (3.2) | | | | | | | | | 7 (2.7) | | | 0 (0.0) | | 0 (0.0) | | 0 (0.0) | | |  |  |
| Obesity | 9 (3.1) | | 0 (0.0) | | | 0 (0.0) | | | 0 (0.0) | | | | | 2 (1.3) | | | | | | | | | 3 (1.1) | | | 1 (0.6) | | 0 (0.0) | | 4 (3.5) | | |  |  |
| Intravenous drugs | 5 (1.7) | | 4 (3.1) | | | 1 (0.6) | | | 5 (1.2) | | | | | 1 (0.6) | | | | | | | | | 4 (1.5) | | | 3 (1.8) | | 1 (2.0) | | 2 (1.7) | | |  |  |
| Inhaled drugs | 56 (19.2) | | 24 (18.5) | | | 32 (19.9) | | | 72 (17.3) | | | | | 22 (14.3) | | | | | | | | | 50 (19.0) | | | 39 (23.8) | | 12 (24.5) | | 27 (23.5) | | |  |  |
| Other drugs | 11 (3.8) | | 8 (6.2) | | | 3 (1.9) | | | 7 (1.7) | | | | | 1 (0.6) | | | | | | | | | 6 (2.3) | | | 2 (1.2) | | 1 (2.0) | | 1 (0.9) | | |  |  |
| Active smoking | 68 (23.4) | | 31 (23.8) | | | 37 (23.0) | | | 110 (26.4) | | | | | 42 (27.3) | | | | | | | | | 68 (25.9) | | | 48 (29.3) | | 13 (26.5) | | 35 (30.4) | | |  |  |
| **Coinfections, n (%)** |  | |  | | |  | | |  | | | | |  | | | | | | | | |  | | |  | |  | |  | | |  |  |
| Chronic Hepatitis B | 13 (4.5) | | 5 (3.8) | | | 8 (5.0) | | | 14 (3.4) | | | | | 3 (1.9) | | | | | | | | | 11 (4.2) | | | 5 (3.0) | | 3 (6.1) | | 2 (1.7) | | |  |  |
| Inactive hepatitis B carrier | 15 (5.2) | | 5 (3.8) | | | 10 (6.2) | | | 18 (4.3) | | | | | 5 (3.2) | | | | | | | | | 13 (4.9) | | | 3 (1.8) | | 1 (2.0) | | 2 (1.7) | | |  |  |
| Hepatitis C | 10 (3.4) | | 2 (1.5) | | | 8 (5.0) | | | 17 (4.1) | | | | | 0 (0.0) | | | | | | | | | 17 (6.5) | | | 12 (7.3) | | 2 (4.1) | | 10 (8.7) | | |  |  |
| None | 256 (88.0) | | 118 (90.8) | | | 134 (83.8) | | | 369 (88.5) | | | | | 146 (94.8) | | | | | | | | | 222 (84.4) | | | 147 (89.6) | | 43 (87.8) | | 101 (87.8) | | |  |  |
| **HIV status and treatment** | |  | | |  | | |  | | | | | | | | | | | | | | | | | | | | | | | | | |  |
| New HIV diagnosis, n (%) | 43 (14.8) | | 24 (18.5) | | | 19 (11.8) | | | 65 (15.6) | | | | | 36 (23.4) | | | | | | | | | 29 (11.0) | | | 21 (12.8) | | 9 (18.4) | | 12 (10.4) | | |  |  |
| Time since diagnosis, years (IQR) | 1.3 (0.2 - 5.8) | | 0.6 (0.1 - 3.8) | | | 1.2 (0.3 - 4.3) | | | 1.1 (0.1 - 6.8) | | | | | 0.2 (0.0 - 2.2) | | | | | | | | | 2.6 (0.3 - 7.5) | | | 3.8 (0.6 - 9.2) | | 1.3 (0.2 - 5.9) | | 5.0 (1.0 - 10.1) | | |  |  |
| **Immunological status at diagnosis** | | | |  | | |  | | | | | | | | | |  | | | | |  | | |  | | | | | | | | |  |
| CD4 cell count at diagnosis, Median (IQR) (n=564) | 108.0 (52.0 - 258.0) | | 69.0 (36.5–131.0) | | | 217.0 (80.0–351.0) | | | 110.0 (43.8 - 243.2) | | | | | 53.0 (21.0–129.5) | | | | | | | | | 184.5 (80.8–348.0) | | | 129.0 (49.0 - 256.0) | | 57.5 (22.0–114.0) | | 218.0 (114.0–381.0) | | |  |  |
| **Stage of infection at diagnosis, n (%)** | | | | | | | | | | | | | | |  | | | | | | | | |  | | |  | |  | |  | | |  |
| Stage I | 17 (5.8) | | 0 (0) | | | 17 (10.6) | | | 29 (7.0) | | | | | 2 (1.3) | | | | | | | | | 27 (10.3) | | | 11 (6.7) | | 0 (0) | | 11 (9.6) | | |  |  |
| Stage II | 52 (17.9) | | 11 (8.5) | | | 41 (25.5) | | | 58 (13.9) | | | | | 8 (5.2) | | | | | | | | | 50 (19) | | | 21 (12.8) | | 1 (2) | | 20 (17.4) | | |  |  |
| Stage III | 160 (55.0) | | 91 (70) | | | 69 (42.9) | | | 220 (52.8) | | | | | 115 (74.7) | | | | | | | | | 105 (39.9) | | | 65 (39.6) | | 35 (71.4) | | 30 (26.1) | | |  |  |
| Unknown | 62 (21.3) | | 28 (21.5) | | | 34 (21.1) | | | 110 (26.4) | | | | | 29 (18.8) | | | | | | | | | 81 (30.8) | | | 67 (40.9) | | 13 (26.5) | | 54 (47) | | |  |  |
| **Immunovirological status at admission** | | | | | | | | | |  | | | | |  | | | | | | | | |  | | |  | |  | |  | | |  |
| CD4 cell count, Median (IQR) (n=723) | 152.0 (53.8 - 342.0) | | 60.0 (26.0–144.0) | | | 265.0 (123.0–423.5) | | | 207.0 (75.2 - 391.0 | | | | | 69.0 (25.0–127.0) | | | | | | | | | 291.0 (154.0–498.5) | | | 216.0 (54.0 - 397.0) | | 51.0 (11.0–129.0) | | 294.5 (166.0–451.2) | | |  |  |
| Stage of infection, n (%) | | | | | | | | | |  | | | | |  | | | | | | | | |  | | |  | |  | |  | | |  |
| Stage I | 27 (9.3) | | 0 (0) | | | 27 (16.8) | | | 62 (14.9) | | | | | 0 (0) | | | | | | | | | 62 (23.6) | | | 18 (11.0) | | 0 (0) | | 18 (15.7) | | |  |  |
| Stage II | 66 (22.7) | | 0 (0) | | | 66 (41) | | | 108 (25.9) | | | | | 0 (0) | | | | | | | | | 108 (41.1) | | | 48 (29.3) | | 0 (0) | | 48 (41.7) | | |  |  |
| Stage III | 192 (66.0) | | 130 (100) | | | 62 (38.5) | | | 243 (58.3) | | | | | 154 (100) | | | | | | | | | 89 (33.8) | | | 94 (57.3) | | 49 (100) | | 45 (39.1) | | |  |  |
| Unknown | 6 (2.1) | | 0 (0) | | | 6 (3.7) | | | 4 (1.0) | | | | | 0 (0) | | | | | | | | | 4 (1.5) | | | 4 (2.4) | | 0 (0) | | 4 (3.5) | | |  |  |
| CD4 Nadir cell count, Median (IQR) (n=723) | 73.0 (29.5 - 193.0) | | 40.0 (20.0–89.5) | | | 126.0 (55.0–330.0) | | | 94.5 (37.2 - 221.8) | | | | | 46.0 (20.8–97.5) | | | | | | | | | 169.0 (78.5–318.5) | | | 89.5 (16.0 - 197.5) | | 22.0 (9.0–71.0) | | 156.0 (47.5–322.2) | | |  |  |
| HIV viral load, n (%) |  | |  | | |  | | |  | | | | |  | | | | | | | | |  | | |  | |  | |  | | |  |  |
| Detectable | 161 (55.3) | | 98 (75.4) | | | 63 (39.1) | | | 230 (55.2) | | | | | 116 (75.3) | | | | | | | | | 114 (43.3) | | | 87 (53.0) | | 37 (75.5) | | 50 (43.5) | | |  |  |
| Undetectable (< 50 copies/mL) | 91 (31.3) | | 18 (13.8) | | | 73 (45.3) | | | 139 (33.3) | | | | | 18 (11.7) | | | | | | | | | 121 (46) | | | 57 (34.8) | | 7 (14.3) | | 50 (43.5) | | |  |  |
| Unknown | 39 (13.4) | | 14 (10.8) | | | 25 (15.5) | | | 48 (11.5) | | | | | 20 (13) | | | | | | | | | 28 (10.6) | | | 20 (12.2) | | 5 (10.2) | | 15 (13) | | |  |  |
| HIV viral load, copies/mL, Median (IQR) (n=478) | 92,045 (4,157 - 432,000) | | 150,584.0 (29,672.5–501,438.2) | | | 32,016.0 (621.0–241,298.5) | | | 91,264 (8,123 - 388,521) | | | | | 196,828.5 (38,266.0–496,396.2) | | | | | | | | | 22,881.5 (787.5–146,632.2) | | | 85,400 (15,246 - 261,150) | | 98,300.0 (29,184.0–361,000.0) | | 48,500.0 (3902.5–201,000.0) | | |  |  |
| **History of ART use, n (%)** | 207 (71.1) | | 77 (59.2) | | | 130 (80.7) | | | 271 (65.0) | | | | | 72 (46.8) | | | | | | | | | 199 (75.7) | | | 121 (73.8) | | 29 (59.2) | | 92 (80.0) | | |  |  |
| Time on ART, years (IQR) | 1.3 (0.4 - 4.6) | | 1.9 (0.3 - 6.9) | | | 1.3 (0.5 - 4.7) | | | 2.2 (0.5 - 5.8) | | | | | 1.2 (0.2 - 4.3) | | | | | | | | | 2.8 (0.7 - 6.5) | | | 4.1 (2.2 - 8.0) | | 3.4 (1.2 - 9.2) | | 4.3 (2.6 - 8.0) | | |  |  |
| **ART Regimen (among treated, n)** | **n=207** | | **n=77** | | | **n=130** | | | **n=271** | |  | |  | | |  | |  |  | | **n=72** | | **n=** **199** | | | **n=** **121** | | **n=** 29 | | **n=** 92 | |  |  |  |
| PI + 2 NRTI: | 73 (35.3) | | 22 (28.6) | | | 51 (39.2 | | | 91 (33.6) | | | | | 20 (27.8 | | | | | | | | | 71 (35.7) | | | 32 (26.4) | | 9 (31.0) | | 23 (25.0) | | |  |  |
| INSTI + 2 NRTI | 60 (29.0) | | 22 (28.6) | | | 38 (29.2 | | | 85 (31.4) | | | | | 26 (36.1 | | | | | | | | | 59 (29.6 | | | 51 (42.1) | | 12 (41.4) | | 39 (42.4) | | |  |  |
| NNRTI + 2 NRTI | 52 (25.1) | | 22 (28.6) | | | 30 (23.1 | | | 85 (31.4) | | | | | 22 (30.6 | | | | | | | | | 63 (31.7 | | | 22 (18.2) | | 5 (17.2 | | 17 (18.5) | | |  |  |
| INSTI + PI | 7 (3.4) | | 2 (2.6) | | | 5 (3.8) | | | 4 (1.5) | | | | | 1 (1.4 | | | | | | | | | **3 (1.5)** | | | 4 (3.3) | | 1 (3.4) | | 3 (3.3) | | |  |  |
| INSTI + PI + 2 NRTI: | 3 (1.4) | | 3 (3.9) | | | 0 (0.0) | | | 2 (0.7) | | | | | 0 (0.0) | | | | | | | | | 2 (1.0) | | | 1 (0.8) | | 0 (0.0) | | 1 (1.1) | |  |  |  |
| Other/unknown | 12 (5.8) | | 6 (7.8) | | | 6 (4.6) | | | 4 (1.5) | | | | | 3 (4.2) | | | | | | | | | 1 (0.5) | | | 11 (9.1) | | 2 (6.9) | | 9 (9.8) | |  |  |  |
| **Adherence (among treated), n (%)** | **n=207** | | **n=77** | | | **n=130** | | | **n=271** | |  | |  | | |  | |  |  | | **n=72** | | **n=** **199** | | | **n=** **121** | | **n=** 29 | | **n=** 92 | |  |  |  |
| Optimal* | 144 (69.6) | | 39 (50.6) | | | 105 (80.8) | | | 201 (74.2) | | | | | 40 (55.6) | | | | | | | | | 161 (80.9) | | | 74 (61.2%) | | 10 (34.5) | | 64 (69.6) | | |  |  |
| Suboptimal** | 63 (30.4) | | 38 (49.4) | | | 25 (19.2) | | | 70 (25.8) | | | | | 32 (44.4) | | | | | | | | | 38 (19.1) | | | 45 (37.2%) | | 19 (65.5) | | 26 (28.3) | | |  |  |
|  |  |  |  |  |  |  |  |  |  |  |  |  |  |  |  |  |  |  |  |  |  |  |  |  |  |  |  |  |  |  |  |  |  | |
| Unknown | 0 (0.0) | | 0 (0) | | | 0 (0) | | | 0 (0.0) | | | | | 0 (0) | | | | | | | | | 0 (0) | | | 0 (0.0) | | 0 (0) | | 2 (2.2) | | |  | |
| **Prophylaxis, n (%)** |  | |  | | |  | | |  | | | | |  | | | | | | | | |  | | |  | |  | |  | | |  | |
| *P. jirovecii* prophylaxis | 101 (34.7) | | 57 (43.8) | | | 44 (27.3) | | | 118 (28.3) | | | | | 58 (37.7) | | | | | | | | | 60 (22.8) | | | 33 (20.1) | | 15 (30.6) | | 18 (15.7) | | |  | |
| MAC prophylaxis | 31 (10.7) | | 18 (13.8) | | | 13 (8.1) | | | 15 (3.6) | | | | | 9 (5.8) | | | | | | | | | 6 (2.3) | | | 2 (1.2) | | 0 (0.0) | | 2 (1.7) | | |  | |
| **Hospitalization** |  | |  | | |  | | |  | | | | |  | | | | | | | | |  | | |  | |  | |  | | |  | |
| Hospital stay, days – median (IQR) | 8.0 (4.0 - 16.0) | | 15.0 (8.2–25.0) | | | 6.0 (3.0–9.0) | | | 8.0 (4.0 - 15.0) | | | | | 13.0 (7.0–27.8) | | | | | | | | | 5.0 (3.0–10.0) | | | 8.0 (3.8 - 15.0) | | 15.0 (8.0–25.0) | | 6.0 (3.0–12.5) | | |  | |
| ICU admission, n (%) | 19 (6.5) | | 14 (10.8) | | | 5 (3.1) | | | 23 (5.5) | | | | | 12 (7.8) | | | | | | | | | 11 (4.2) | | | 17 (10.4 | | 12 (24.5) | | 5 (4.3) | | |  | |
| ICU stay, days Median (IQR) | 4.0 (2.5 - 9.5) | | 4.0 (3.0–9.8) | | | 6.0 (1.0–7.0) | | | 9.0 (6.5 - 13.0) | | | | | 8.0 (5.8–11.0) | | | | | | | | | 12.0 (7.5–13.0) | | | 4.0 (3.0 - 6.0) | | 4.5 (3.0–6.2) | | 4.0 (3.0–6.0) | | |  | |
| Readmission within 30 days, n (%) | 39 (13.4) | | 26 (20.0) | | | 13 (8.1) | | | 55 (13.2) | | | | | 30 (19.5) | | | | | | | | | 25 (9.5) | | | 17 (10.4 | | 7 (14.3) | | 10 (8.7) | | |  | |
| In-hospital Mortality, n (%) | 13 (4.5) | | 10 (7.7) | | | 3 (1.9) | | | 15 (3.6) | | | | | 10 (6.5) | | | | | | | | | 5 (1.9) | | | 9 (5.5) | | 7 (14.3) | | 2 (1.7) | | |  | |

**Abbreviations:** ART, Antiretroviral therapy; COPD, Chronic Obstructive Pulmonary Disease; HTN, Hypertension; ICU, Intensive Care Unit; INSTI, Integrase strand transfer inhibitor; IQR, Interquartile range; MAC, *Mycobacterium avium* complex; NNRTI, Non-nucleoside reverse transcriptase inhibitor; NRTI, Nucleoside/nucleotide reverse transcriptase inhibitor; PI, Protease inhibitor; T2DM, Type 2 Diabetes Mellitus. **Note:** * Optimal adherence: At least 95% of doses taken and no lapse of > 48 hrs. ** Suboptimal adherence: Less than 95% of doses taken and/or lapse of > 48 hrs. **Pre-COVID-19:** Admissions before March 1, 2020. **During COVID-19:** Admissions from March 1, 2020, to May 5, 2023 (date when WHO declared the end of the global health emergency). **Post-COVID-19:** Admissions after May 5, 2023.
