# Supplementary material for: Causes of hospitalization among people living with HIV in a tertiary-care hospital in Bogotá, Colombia, 2018–2024
Source: PLoS One. 2026 Sep 30;21(9):e0357788. doi: 10.1371/journal.pone.0357788 (PMC13626370; doi:10.1371/journal.pone.0357788)
Supplement: Supplementary file 3 [file pone.0357788.s007.docx]

**Response to Reviewers**

Manuscript ID: PONE-D-25-67550

Title: Causes of hospitalization among people living with HIV in a tertiary-care hospital in Bogotá, Colombia, 2018–2024

We thank the Academic Editor and Reviewer for their careful evaluation of our manuscript. We have addressed all comments and revised the manuscript accordingly. Changes are shown in the marked-up manuscript, and a clean revised version is also provided.

### **Journal Requirement 1**

**Comment:**
Please ensure that your manuscript meets PLOS ONE's style requirements, including those for file naming.

**Response:**
Thank you. We reviewed the PLOS ONE style and file-naming requirements and revised the manuscript and Supporting Information files accordingly. The Supporting Information captions are included at the end of the revised manuscript, after the References section. The corresponding files have been named consistently with their citations and captions in the manuscript: S1_Table.docx, S2_Table.docx, S1_Fig.tif, S2_Fig.tif, S3_Fig.tif, and S1_Dataset.xlsx.

### **Journal Requirement 2**

**Comment:**
We are unable to open your Supporting Information file “S1_Table.docx, S2_Fig.tiff and S1_Fig.tiff.” Please kindly revise as necessary and re-upload.

**Response:**
Thank you for bringing this issue to our attention. We recreated the three Supporting Information files and verified that they can be opened correctly. The S1 Table was saved as a new standalone DOCX file without the supporting figures embedded within it. The S1 and S2 Figures were re-encoded as standard RGB TIFF files with LZW compression and a resolution of 300 dpi. Additional white margins were included around the figures to prevent the titles, labels, and legends from being cropped. The corrected files have been re-uploaded as S1_Table.docx, S1_Fig.tif, and S2_Fig.tif.

### **Journal Requirement #3: Identifying information in the S1 Dataset**

**Comment:**
We note that there is identifying data in the Supporting Information file “S1_Dataset.xlsx.” Please remove or anonymize all personal information, ensure that the data shared are in accordance with participant consent, and re-upload a fully anonymized dataset.

**Response:**
We thank the Editorial Office for identifying this concern. We carefully reviewed and further de-identified the S1 Dataset in accordance with the PLOS Data Policy for human research participant data.

We deleted all columns containing specific calendar dates, including the dates of HIV diagnosis, hospital admission, ART initiation, hospital discharge, death, ICU admission, and ICU discharge. These columns were permanently removed and were not hidden.

To preserve the analytical value of the dataset, relevant dates were replaced with derived time intervals expressed in whole days, including hospital length of stay, time from HIV diagnosis to admission, time receiving ART before admission, time from hospital admission to ICU admission, ICU length of stay, and time from hospital admission to death. No calendar dates remain in the revised dataset.

We also deleted the following five free-text variables because they contained detailed clinical descriptions that could potentially contribute to indirect identification:

- other_comorbidity
- neoplasm_cause_specify
- other_cause
- neoplasm_condition_specify
- other_condition

In addition, the original record identifier was replaced. The dataset rows were randomly reordered, and a new sequential record_id ranging from 1 to 872 was generated. This new identifier has no correspondence with the original REDCap identifier, hospital identifier, or medical record number.

The remaining dataset contains no names, initials, addresses, contact information, IP addresses, geographic information, medical record numbers, or other direct personal identifiers. Age is reported only in completed years. We also verified that there are no hidden rows or columns containing identifying information.

The revised data dictionary was updated accordingly, and the de-identified dataset has been re-uploaded as **S1_Dataset.xlsx**.

The study was approved by the Institutional Research Ethics Committee of Hospital Universitario San Ignacio and Pontificia Universidad Javeriana, Bogotá, Colombia (protocol approval No. 2025/052; approval letter No. FM-CIE-0316-25).

**Comments to the Author**

1. Is the manuscript technically sound, and do the data support the conclusions?

The manuscript must describe a technically sound piece of scientific research with data that supports the conclusions. Experiments must have been conducted rigorously, with appropriate controls, replication, and sample sizes. The conclusions must be drawn appropriately based on the data presented.

Reviewer #1: Yes

2. Has the statistical analysis been performed appropriately and rigorously?

Reviewer #1: Yes

3. Have the authors made all data underlying the findings in their manuscript fully available?

The [PLOS Data policy](https://nam10.safelinks.protection.outlook.com/?url=http%3A%2F%2Ftrack.editorialmanager.com%2FCL0%2Fhttp%3A%252F%252Fwww.plosone.org%252Fstatic%252Fpolicies.action%2523sharing%2F1%2F010f019f11e8da40-58382a09-690b-46fb-932a-aae596173b4f-000000%2F-juRRg4W2fKRIQKsxBAzAosZpohZU6kkgBvbbFTKpXw%3D258&data=05%7C02%7Cdlrico%40javeriana.edu.co%7Cd3eff6ae789a4e2e6afe08ded5e3c0f1%7Cdaf7990e8a3f409c9b762a5475098000%7C0%7C0%7C639183371380316354%7CUnknown%7CTWFpbGZsb3d8eyJFbXB0eU1hcGkiOnRydWUsIlYiOiIwLjAuMDAwMCIsIlAiOiJXaW4zMiIsIkFOIjoiTWFpbCIsIldUIjoyfQ%3D%3D%7C40000%7C%7C%7C&sdata=tG%2BTkp6pXwj0OWhQnmusDkv0GHu8rdz3PEX3uyCME4o%3D&reserved=0) requires authors to make all data underlying the findings described in their manuscript fully available without restriction, with rare exception (please refer to the Data Availability Statement in the manuscript PDF file). The data should be provided as part of the manuscript or its supporting information, or deposited to a public repository. For example, in addition to summary statistics, the data points behind means, medians and variance measures should be available. If there are restrictions on publicly sharing data—e.g. participant privacy or use of data from a third party—those must be specified.

Reviewer #1: Yes

4. Is the manuscript presented in an intelligible fashion and written in standard English?

PLOS ONE does not copyedit accepted manuscripts, so the language in submitted articles must be clear, correct, and unambiguous. Any typographical or grammatical errors should be corrected at revision, so please note any specific errors here.

Reviewer #1: Yes

5. Review Comments to the Author

Please use the space provided to explain your answers to the questions above. You may also include additional comments for the author, including concerns about dual publication, research ethics, or publication ethics. (Please upload your review as an attachment if it exceeds 20,000 characters)

Reviewer #1:

Dear Editor,
I would like to thank you for the opportunity to review this manuscript. The study addresses an important and timely topic regarding hospitalization patterns among people living with HIV in a tertiary care hospital in Bogotá, Colombia, particularly in the context of the COVID-19 pandemic. Overall, the manuscript is well structured, clinically relevant, and contributes valuable evidence to HIV epidemiology and health services research.
However, several important issues related to methodological transparency, clarity of definitions, and completeness of reporting require revision in order to improve interpretability, reproducibility, and scientific rigor.
The specific comments are outlined below.

**Introduction**

1. A more integrated epidemiological overview of HIV in Colombia is needed in the introduction. In particular, a unified presentation of key indicators such as prevalence, incidence, mortality, and hospitalization rates is essential. At present, these indicators are reported in a fragmented manner, which limits a comprehensive understanding of the national burden of disease.
Methods Section:

**Response:** We thank the reviewer for this important suggestion. We revised the Introduction to provide a more integrated overview of the HIV epidemiological situation in Colombia. The revised paragraph now presents national data on HIV prevalence, incidence, all-cause mortality, ART coverage, and viral suppression in a unified manner. We also explicitly acknowledge that a national hospitalization rate for people living with HIV has not been published and summarize the most comprehensive available evidence on causes of hospitalization in Colombia. This revision provides a clearer overview of the national burden of HIV and better highlights the evidence gap addressed by our study.

The corresponding changes have been incorporated into the Introduction.

2. It is necessary to explicitly address the implications of excluding patients transferred within 72 hours in the limitations section. This decision may introduce selection bias and reduce the generalizability of the study findings.

**Response:** We thank the reviewer for this important observation. We agree that excluding hospitalization episodes involving transfer to another institution within the first 72 hours may have introduced selection bias and limited the generalizability of our findings. This criterion was applied to ensure complete ascertainment of clinical events and outcomes, including total length of stay, ICU admission, in-hospital mortality, and 30-day mortality, which could not be reliably assessed once care continued at another institution. Nevertheless, we acknowledge that early transfers—often driven in our setting by insurance-related administrative processes rather than clinical severity—may represent a distinct clinical profile. We have therefore added this issue to the limitations section, noting that the exclusion may have reduced representation of the full clinical spectrum. However, given the relatively small proportion of excluded cases (7%), we expect any resulting effect on our main findings to have been modest.

The corresponding changes have been incorporated into the Limitations section.

3. A brief description of Hospital Universitario San Ignacio (HUSI), including its role as a tertiary referral center and its contribution to HIV-related hospitalizations in Bogotá, should be provided. In addition, a short description of Bogotá (population size and general characteristics of the healthcare system) would help international readers better understand the study setting.

**Response:** We thank the reviewer for this helpful suggestion. We expanded the Methods section to provide additional context on both the study setting and the role of HUSI in HIV care. We added a brief description of Bogotá, including its population size and the general organization of the Colombian health insurance system. We also clarified that HUSI is a high-complexity teaching and referral center and that its Infectious Diseases service includes a dedicated HIV care program serving people living with HIV affiliated with multiple health insurers, supporting its role as a referral center for HIV care in Bogotá and surrounding areas.

The corresponding changes have been incorporated into the Methods section (“Study design and population”).

4. The description of data extraction and entry into the REDCap database lacks clarity. It is not specified who performed data entry or whether standardized procedures or quality control measures were implemented. Furthermore, it is unclear whether sociodemographic variables (including socioeconomic status and sexual orientation) were obtained from electronic medical records or through direct patient interviews. If these variables were extracted from clinical records, the authors should clarify who originally recorded them and how data reliability and completeness were ensured.

**Response:** We thank the reviewer for this important comment. We have revised the Methods section to clarify the data extraction and quality-control procedures. Clinical and sociodemographic data were extracted from the electronic medical records by trained members of the research team, including physicians and residents, and entered into a structured REDCap database using a predefined instrument with controlled response options and built-in field-validation rules. As a quality-control measure, a sample of records was independently cross-checked against the source medical records by a second reviewer, and identified discrepancies were corrected by returning to the primary source. We also clarified that sociodemographic variables, including educational level and sexual orientation, were obtained exclusively from the electronic medical records and had been documented during routine clinical care by members of the interdisciplinary care team. No direct patient interviews were conducted for this study, and variables not documented in the medical record were treated as missing.

The corresponding changes have been incorporated into the Methods section (“Data processing and collection”).

5. A separate subsection defining and operationalizing study variables should be added. In particular, socioeconomic status and sexual orientation should be clearly defined and categorized, and their measurement procedures should be explicitly described.

**Response:**

We thank the reviewer for highlighting this point. We clarified the description of the sociodemographic variables and noted that the variable collected in our database was educational level, rather than socioeconomic status as stated in the original version; the manuscript has been corrected accordingly. Although socioeconomic status was not directly measured, educational level was available and was used as a proxy for socioeconomic position. We also added a separate “Study variables” subsection to the Methods that explicitly defines and categorizes the main study variables, including sexual orientation and educational level, and describes how they were measured. The corresponding changes have been incorporated into the Methods section (“Study variables” subsection).

6. The assessment of antiretroviral therapy (ART) adherence based on self-report requires further clarification. It should be specified whether this information was collected by research staff or clinical personnel, and whether it was obtained from medical records or direct patient interviews. If recorded in clinical charts, the manuscript should clarify how this information was originally collected and the extent to which it can be considered reliable, given the known limitations of self-reported adherence data.

**Response:** We thank the reviewer for this important observation. We clarified that ART adherence was based on patient self-report elicited by the treating clinical team during routine care and documented in the electronic medical record; this information was subsequently abstracted by the research team. No adherence interviews were conducted specifically for this study. We also clarified the operational definition used for adherence and explicitly acknowledged the limitations of self-reported adherence, including recall and social-desirability bias, which may lead to overestimation. In addition, we noted that admission HIV viral load was available as an objective complementary marker of treatment response.

The corresponding changes have been incorporated into the Methods and Limitations sections.

7. The statistical analysis section does not adequately describe the handling of missing data. The authors should report the extent of missingness for each variable, specify the methods used to handle missing data (e.g., complete-case analysis or imputation).

**Response:**
 We thank the reviewer for this important observation. We have expanded the Statistical Analysis section to clarify the handling of missing data. No imputation was performed, and analyses were conducted using available data for each variable. Variables that were conditional by design were analyzed within their applicable subgroups and were not considered missing when not applicable. Values recorded as unknown or not documented were retained as separate categories in descriptive analyses. In addition, the extent of missingness and the corresponding analytic denominators are now explicitly reported in Table 1, with Table 2 referring to these data.

These changes have been incorporated into the Statistical Analysis section and the notes to Tables 1 and 2.

**Results Section:**

8. “In total, 2,109 records from the hospital administrative database were reviewed, of which 857 were related to HIV diagnoses or AIDS-defining conditions.” At this stage of the manuscript, it remains unclear whether all patients with confirmed HIV infection were included regardless of admission diagnosis (both AIDS-defining and non–AIDS-defining conditions), or whether inclusion was restricted to HIV/AIDS-related admissions only. This ambiguity regarding inclusion criteria should be clearly clarified either in the Methods section or at the beginning of the Results section to ensure transparency of the study population definition.

**Response:**
We thank the reviewer for highlighting this important point. We agree that the original wording could be misinterpreted as restricting inclusion to HIV/AIDS-related admissions. We have clarified that eligibility was based on HIV status rather than on the reason for admission. Specifically, we included all hospitalization episodes of adults with confirmed HIV infection, regardless of whether the admission was due to an AIDS-defining or a non–AIDS-defining condition.

To identify potentially eligible episodes, we first performed a broad administrative query using infectious-disease ICD-10 codes. This case-finding strategy intentionally included diagnoses beyond HIV and therefore retrieved records that did not correspond to confirmed HIV infection. Each retrieved record was reviewed to confirm HIV infection. Eligibility was subsequently verified by cross-referencing the Infectious Diseases service database, which also identified additional eligible hospitalization episodes not captured through administrative coding.

We have clarified this in the Methods and at the beginning of the Results section. To further improve transparency regarding the case-identification and inclusion process, we also added a study flow diagram as S3 Fig.

9. The variable related to psychoactive substance use requires clearer definition and methodological description in the Methods section. It is not specified whether this information was obtained through self-report, extracted from electronic medical records, or based on clinical assessment. Furthermore, the criteria used to classify different types of substance use (e.g., smoking, inhalants, and other substances) should be explicitly described, along with clarification of data validity and reliability.

**Response:**

We thank the reviewer for this observation. We have clarified in the Methods that psychoactive substance use was based primarily on patient self-report documented during routine clinical care, including the structured psychosocial assessment performed by the social work team, and subsequently abstracted from the electronic medical record. Substance use was recorded as a multiple-response variable using predefined categories. We also clarified in the table note that these categories were non-mutually exclusive. No study-specific interviews or toxicological testing were performed. We also acknowledged the potential for underreporting or misclassification in the Limitations section.
