# Supplementary material for: Causes of hospitalization among people living with HIV in a tertiary-care hospital in Bogotá, Colombia, 2018–2024
Source: PLoS One. 2026 Sep 30;21(9):e0357788. doi: 10.1371/journal.pone.0357788 (PMC13626370; doi:10.1371/journal.pone.0357788)
Supplement: Peer review history

# Peer review: Decision Letter 0

**DOI:** 10.1371/journal.pone.0357788.r001

**Authors:** Felix Bongomin

29 Jun 2026

Dear Dr. Valderrama-Beltrán,

Thank you for submitting your manuscript to PLOS ONE. After careful consideration, we feel that it has merit but does not fully meet PLOS ONE’s publication criteria as it currently stands. Therefore, we invite you to submit a revised version of the manuscript that addresses the points raised during the review process.

Please submit your revised manuscript by Aug 13 2026 11:59PM. If you will need more time than this to complete your revisions, please reply to this message or contact the journal office at plosone@plos.org. When you're ready to submit your revision, log on to https://www.editorialmanager.com/pone/ and select the 'Submissions Needing Revision' folder to locate your manuscript file.

- A letter that responds to each point raised by the academic editor and reviewer(s). You should upload this letter as a separate file labeled 'Response to Reviewers'.
- A marked-up copy of your manuscript that highlights changes made to the original version. You should upload this as a separate file labeled 'Revised Manuscript with Track Changes'.
- An unmarked version of your revised paper without tracked changes. You should upload this as a separate file labeled 'Manuscript'.

If you would like to make changes to your financial disclosure, please include your updated statement in your cover letter. Guidelines for resubmitting your figure files are available below the reviewer comments at the end of this letter.

If applicable, we recommend that you deposit your laboratory protocols in protocols.io to enhance the reproducibility of your results. Protocols.io assigns your protocol its own identifier (DOI) so that it can be cited independently in the future. For instructions see: https://journals.plos.org/plosone/s/submission-guidelines#loc-laboratory-protocols. Additionally, PLOS ONE offers an option for publishing peer-reviewed Lab Protocol articles, which describe protocols hosted on protocols.io. Read more information on sharing protocols at https://plos.org/protocols?utm_medium=editorial-email&utm_source=authorletters&utm_campaign=protocols.

As the corresponding author, your ORCID iD is verified in the submission system and will appear in the published article. PLOS supports the use of ORCID, and we encourage all coauthors to register for an ORCID iD and use it as well. Please encourage your coauthors to verify their ORCID iD within the submission system before final acceptance, as unverified ORCID iDs will not appear in the published article. *Only*  the individual author can complete the verification step; PLOS staff *cannot*  verify ORCID iDs on behalf of authors.

We look forward to receiving your revised manuscript.

Kind regards,

Felix Bongomin, MB ChB, Ph.D., FECMM

Academic Editor

PLOS One

Journal Requirements:

When submitting your revision, we need you to address these additional requirements.

1.Please ensure that your manuscript meets PLOS ONE's style requirements, including those for file naming. The PLOS ONE style templates can be found at

https://journals.plos.org/plosone/s/file?id=wjVg/PLOSOne_formatting_sample_main_body.pdf and

https://journals.plos.org/plosone/s/file?id=ba62/PLOSOne_formatting_sample_title_authors_affiliations.pdf

2. We are unable to open your Supporting Information file “S1_Table.docx, S2_Fig.tiff and S1_Fig.tiff” Please kindly revise as necessary and re-upload.

3. We note that there is identifying data in the Supporting Information file “S1_Dataset.xlsx” Due to the inclusion of these potentially identifying data, we have removed this file from your file inventory. Prior to sharing human research participant data, authors should consult with an ethics committee to ensure data are shared in accordance with participant consent and all applicable local laws.

Data sharing should never compromise participant privacy. It is therefore not appropriate to publicly share personally identifiable data on human research participants. The following are examples of data that should not be shared:

-Name, initials, physical address

-Ages more specific than whole numbers

-Internet protocol (IP) address

-Specific dates (birth dates, death dates, examination dates, etc.)

-Contact information such as phone number or email address

-Location data

-ID numbers that seem specific (long numbers, include initials, titled “Hospital ID”) rather than random (small numbers in numerical order)

Data that are not directly identifying may also be inappropriate to share, as in combination they can become identifying. For example, data collected from a small group of participants, vulnerable populations, or private groups should not be shared if they involve indirect identifiers (such as sex, ethnicity, location, etc.) that may risk the identification of study participants.

Additional guidance on preparing raw data for publication can be found in our Data Policy (https://journals.plos.org/plosone/s/data-availability#loc-human-research-participant-data-and-other-sensitive-data) and in the following article: http://www.bmj.com/content/340/bmj.c181.long.

Please remove or anonymize all personal information (<specific identifying information in file to be removed>), ensure that the data shared are in accordance with participant consent, and re-upload a fully anonymized data set. Please note that spreadsheet columns with personal information must be removed and not hidden as all hidden columns will appear in the published file.

4. If the reviewer comments include a recommendation to cite specific previously published works, please review and evaluate these publications to determine whether they are relevant and should be cited. There is no requirement to cite these works unless the editor has indicated otherwise.

[Note: HTML markup is below. Please do not edit.]

Reviewer's Responses to Questions

**Comments to the Author**

1. Is the manuscript technically sound, and do the data support the conclusions?

Reviewer #1: Yes

**********

2. Has the statistical analysis been performed appropriately and rigorously?

Reviewer #1: Yes

**********

3. Have the authors made all data underlying the findings in their manuscript fully available?

The PLOS Data policy

Reviewer #1: Yes

**********

4. Is the manuscript presented in an intelligible fashion and written in standard English?

Reviewer #1: Yes

**********

Reviewer #1: Dear Editor,

I would like to thank you for the opportunity to review this manuscript. The study addresses an important and timely topic regarding hospitalization patterns among people living with HIV in a tertiary care hospital in Bogotá, Colombia, particularly in the context of the COVID-19 pandemic. Overall, the manuscript is well structured, clinically relevant, and contributes valuable evidence to HIV epidemiology and health services research.

However, several important issues related to methodological transparency, clarity of definitions, and completeness of reporting require revision in order to improve interpretability, reproducibility, and scientific rigor.

The specific comments are outlined below.

Introduction

1. A more integrated epidemiological overview of HIV in Colombia is needed in the introduction. In particular, a unified presentation of key indicators such as prevalence, incidence, mortality, and hospitalization rates is essential. At present, these indicators are reported in a fragmented manner, which limits a comprehensive understanding of the national burden of disease.

Methods Section:

2. It is necessary to explicitly address the implications of excluding patients transferred within 72 hours in the limitations section. This decision may introduce selection bias and reduce the generalizability of the study findings.

3. A brief description of Hospital Universitario San Ignacio (HUSI), including its role as a tertiary referral center and its contribution to HIV-related hospitalizations in Bogotá, should be provided. In addition, a short description of Bogotá (population size and general characteristics of the healthcare system) would help international readers better understand the study setting.

4. The description of data extraction and entry into the REDCap database lacks clarity. It is not specified who performed data entry or whether standardized procedures or quality control measures were implemented. Furthermore, it is unclear whether sociodemographic variables (including socioeconomic status and sexual orientation) were obtained from electronic medical records or through direct patient interviews. If these variables were extracted from clinical records, the authors should clarify who originally recorded them and how data reliability and completeness were ensured.

5. A separate subsection defining and operationalizing study variables should be added. In particular, socioeconomic status and sexual orientation should be clearly defined and categorized, and their measurement procedures should be explicitly described.

6. The assessment of antiretroviral therapy (ART) adherence based on self-report requires further clarification. It should be specified whether this information was collected by research staff or clinical personnel, and whether it was obtained from medical records or direct patient interviews. If recorded in clinical charts, the manuscript should clarify how this information was originally collected and the extent to which it can be considered reliable, given the known limitations of self-reported adherence data.

7. The statistical analysis section does not adequately describe the handling of missing data. The authors should report the extent of missingness for each variable, specify the methods used to handle missing data (e.g., complete-case analysis or imputation).

Results Section:

8. “In total, 2,109 records from the hospital administrative database were reviewed, of which 857 were related to HIV diagnoses or AIDS-defining conditions.” At this stage of the manuscript, it remains unclear whether all patients with confirmed HIV infection were included regardless of admission diagnosis (both AIDS-defining and non–AIDS-defining conditions), or whether inclusion was restricted to HIV/AIDS-related admissions only. This ambiguity regarding inclusion criteria should be clearly clarified either in the Methods section or at the beginning of the Results section to ensure transparency of the study population definition.

9. The variable related to psychoactive substance use requires clearer definition and methodological description in the Methods section. It is not specified whether this information was obtained through self-report, extracted from electronic medical records, or based on clinical assessment. Furthermore, the criteria used to classify different types of substance use (e.g., smoking, inhalants, and other substances) should be explicitly described, along with clarification of data validity and reliability.

**********

what does this mean?). If published, this will include your full peer review and any attached files.

If you choose “no”, your identity will remain anonymous but your review may still be made public.

**Do you want your identity to be public for this peer review?**  For information about this choice, including consent withdrawal, please see our Privacy Policy

Reviewer #1: No

**********

[NOTE: If reviewer comments were submitted as an attachment file, they will be attached to this email and accessible via the submission site. Please log into your account, locate the manuscript record, and check for the action link "View Attachments". If this link does not appear, there are no attachment files.]

To ensure your figures meet our technical requirements, please review our figure guidelines: https://journals.plos.org/plosone/s/figures

You may also use PLOS’s free figure tool, NAAS, to help you prepare publication quality figures: https://journals.plos.org/plosone/s/figures#loc-tools-for-figure-preparation.

NAAS will assess whether your figures meet our technical requirements by comparing each figure against our figure specifications.

# Author comment: Author response to Decision Letter 1

**DOI:** 10.1371/journal.pone.0357788.r002

13 Aug 2026

Response to Reviewers

Manuscript ID: PONE-D-25-67550

Title: Causes of hospitalization among people living with HIV in a tertiary-care hospital in Bogotá, Colombia, 2018–2024

We thank the Academic Editor and Reviewer for their careful evaluation of our manuscript. We have addressed all comments and revised the manuscript accordingly. Changes are shown in the marked-up manuscript, and a clean revised version is also provided.

Journal Requirement 1

Comment:

Please ensure that your manuscript meets PLOS ONE's style requirements, including those for file naming.

Response:

Thank you. We reviewed the PLOS ONE style and file-naming requirements and revised the manuscript and Supporting Information files accordingly. The Supporting Information captions are included at the end of the revised manuscript, after the References section. The corresponding files have been named consistently with their citations and captions in the manuscript: S1_Table.docx, S2_Table.docx, S1_Fig.tif, S2_Fig.tif, S3_Fig.tif, and S1_Dataset.xlsx.

Journal Requirement 2

Comment:

We are unable to open your Supporting Information file “S1_Table.docx, S2_Fig.tiff and S1_Fig.tiff.” Please kindly revise as necessary and re-upload.

Response:

Thank you for bringing this issue to our attention. We recreated the three Supporting Information files and verified that they can be opened correctly. The S1 Table was saved as a new standalone DOCX file without the supporting figures embedded within it. The S1 and S2 Figures were re-encoded as standard RGB TIFF files with LZW compression and a resolution of 300 dpi. Additional white margins were included around the figures to prevent the titles, labels, and legends from being cropped. The corrected files have been re-uploaded as S1_Table.docx, S1_Fig.tif, and S2_Fig.tif.

Journal Requirement #3: Identifying information in the S1 Dataset

Comment:

We note that there is identifying data in the Supporting Information file “S1_Dataset.xlsx.” Please remove or anonymize all personal information, ensure that the data shared are in accordance with participant consent, and re-upload a fully anonymized dataset.

Response:

We thank the Editorial Office for identifying this concern. We carefully reviewed and further de-identified the S1 Dataset in accordance with the PLOS Data Policy for human research participant data.

We deleted all columns containing specific calendar dates, including the dates of HIV diagnosis, hospital admission, ART initiation, hospital discharge, death, ICU admission, and ICU discharge. These columns were permanently removed and were not hidden.

To preserve the analytical value of the dataset, relevant dates were replaced with derived time intervals expressed in whole days, including hospital length of stay, time from HIV diagnosis to admission, time receiving ART before admission, time from hospital admission to ICU admission, ICU length of stay, and time from hospital admission to death. No calendar dates remain in the revised dataset.

We also deleted the following five free-text variables because they contained detailed clinical descriptions that could potentially contribute to indirect identification:

● other_comorbidity

● neoplasm_cause_specify

● other_cause

● neoplasm_condition_specify

● other_condition

In addition, the original record identifier was replaced. The dataset rows were randomly reordered, and a new sequential record_id ranging from 1 to 872 was generated. This new identifier has no correspondence with the original REDCap identifier, hospital identifier, or medical record number.

The remaining dataset contains no names, initials, addresses, contact information, IP addresses, geographic information, medical record numbers, or other direct personal identifiers. Age is reported only in completed years. We also verified that there are no hidden rows or columns containing identifying information.

The revised data dictionary was updated accordingly, and the de-identified dataset has been re-uploaded as S1_Dataset.xlsx.

The study was approved by the Institutional Research Ethics Committee of Hospital Universitario San Ignacio and Pontificia Universidad Javeriana, Bogotá, Colombia (protocol approval No. 2025/052; approval letter No. FM-CIE-0316-25).

Comments to the Author

1. Is the manuscript technically sound, and do the data support the conclusions?

The manuscript must describe a technically sound piece of scientific research with data that supports the conclusions. Experiments must have been conducted rigorously, with appropriate controls, replication, and sample sizes. The conclusions must be drawn appropriately based on the data presented.

Reviewer #1: Yes

________________________________________

2. Has the statistical analysis been performed appropriately and rigorously?

Reviewer #1: Yes

________________________________________

3. Have the authors made all data underlying the findings in their manuscript fully available?

The PLOS Data policy requires authors to make all data underlying the findings described in their manuscript fully available without restriction, with rare exception (please refer to the Data Availability Statement in the manuscript PDF file). The data should be provided as part of the manuscript or its supporting information, or deposited to a public repository. For example, in addition to summary statistics, the data points behind means, medians and variance measures should be available. If there are restrictions on publicly sharing data—e.g. participant privacy or use of data from a third party—those must be specified.

Reviewer #1: Yes

________________________________________

4. Is the manuscript presented in an intelligible fashion and written in standard English?

PLOS ONE does not copyedit accepted manuscripts, so the language in submitted articles must be clear, correct, and unambiguous. Any typographical or grammatical errors should be corrected at revision, so please note any specific errors here.

Reviewer #1: Yes

________________________________________

5. Review Comments to the Author

Please use the space provided to explain your answers to the questions above. You may also include additional comments for the author, including concerns about dual publication, research ethics, or publication ethics. (Please upload your review as an attachment if it exceeds 20,000 characters)

Reviewer #1:

Dear Editor,

I would like to thank you for the opportunity to review this manuscript. The study addresses an important and timely topic regarding hospitalization patterns among people living with HIV in a tertiary care hospital in Bogotá, Colombia, particularly in the context of the COVID-19 pandemic. Overall, the manuscript is well structured, clinically relevant, and contributes valuable evidence to HIV epidemiology and health services research.

However, several important issues related to methodological transparency, clarity of definitions, and completeness of reporting require revision in order to improve interpretability, reproducibility, and scientific rigor.

The specific comments are outlined below.

Introduction

1. A more integrated epidemiological overview of HIV in Colombia is needed in the introduction. In particular, a unified presentation of key indicators such as prevalence, incidence, mortality, and hospitalization rates is essential. At present, these indicators are reported in a fragmented manner, which limits a comprehensive understanding of the national burden of disease.

Methods Section:

Response: We thank the reviewer for this important suggestion. We revised the Introduction to provide a more integrated overview of the HIV epidemiological situation in Colombia. The revised paragraph now presents national data on HIV prevalence, incidence, all-cause mortality, ART coverage, and viral suppression in a unified manner. We also explicitly acknowledge that a national hospitalization rate for people living with HIV has not been published and summarize the most comprehensive available evidence on causes of hospitalization in Colombia. This revision provides a clearer overview of the national burden of HIV and better highlights the evidence gap addressed by our study.

The corresponding changes have been incorporated into the Introduction on page 4, lines 79–93.

2. It is necessary to explicitly address the implications of excluding patients transferred within 72 hours in the limitations section. This decision may introduce selection bias and reduce the generalizability of the study findings.

Response: We thank the reviewer for this important observation. We agree that excluding hospitalization episodes involving transfer to another institution within the first 72 hours may have introduced selection bias and limited the generalizability of our findings. This criterion was applied to ensure complete ascertainment of clinical events and outcomes, including total length of stay, ICU admission, in-hospital mortality, and 30-day mortality, which could not be reliably assessed once care continued at another institution. Nevertheless, we acknowledge that early transfers—often driven in our setting by insurance-related administrative processes rather than clinical severity—may represent a distinct clinical profile. We have therefore added this issue to the limitations section, noting that the exclusion may have reduced representation of the full clinical spectrum. However, given the relatively small proportion of excluded cases (7%), we expect any resulting effect on our main findings to have been modest.

The corresponding changes have been incorporated into the Limitations section on page 29, lines 515–521.

3. A brief description of Hospital Universitario San Ignacio (HUSI), including its role as a tertiary referral center and its contribution to HIV-related hospitalizations in Bogotá, should be provided. In addition, a short description of Bogotá (population size and general characteristics of the healthcare system) would help international readers better understand the study setting.

Response: We thank the reviewer for this helpful suggestion. We expanded the Methods section to provide additional context on both the study setting and the role of HUSI in HIV care. We added a brief description of Bogotá, including its population size and the general organization of the Colombian health insurance system. We also clarified that HUSI is a high-complexity teaching and referral center and that its Infectious Diseases service includes a dedicated HIV care program serving people living with HIV affiliated with multiple health insurers, supporting its role as a referral center for HIV care in Bogotá and surrounding areas.

The corresponding changes have been incorporated into the Methods section (“Study design and population”) on page 5, lines 101–111.

4. The description of data extraction and entry into the REDCap database lacks clarity. It is not specified who performed data entry or whether standardized procedures or quality control measures were implemented. Furthermore, it is unclear whether sociodemographic variables (including socioeconomic status and sexual orientation) were obtained from electronic medical records or through direct patient interviews. If these variables were extracted from clinical records, the authors should clarify who originally recorded them and how data reliability and completeness were ensured.

Response: We thank the reviewer for this important comment. We have revised the Methods section to clarify the data extraction and quality-control procedures. Clinical and sociodemographic data were extracted from the electronic medical records by trained members of the research team, including physicians and residents, and entered into a structured REDCap database using a predefined instrument with controlled response options and built-in field-validation rules. As a quality-control measure, a sample of records was independently cross-checked against the source medical records by a second reviewer, and identified discrepancies were corrected by returning to the primary source. We also clarified that sociodemographic variables, including educational level and sexual orientation, were obtained exclusively from the electronic medical records and had been documented during routine clinical care by members of the interdisciplinary care team. No direct patient interviews were conducted for this study, and variables not documented in the medical record were treated as missing.

The corresponding changes have been incorporated into the Methods section (“Data processing and collection”) on pages 6–7, lines 134–144.

5. A separate subsection defining and operationalizing study variables should be added. In particular, socioeconomic status and sexual orientation should be clearly defined and categorized, and their measurement procedures should be explicitly described.

Response:

We thank the reviewer for highlighting this point. We clarified the description of the sociodemographic variables and noted that the variable collected in our database was educational level, rather than socioeconomic status as stated in the original version; the manuscript has been corrected accordingly. Although socioeconomic status was not directly measured, educational level was available and was used as a proxy for socioeconomic position. We also added a separate “Study variables” subsection to the Methods that explicitly defines and categorizes the main study variables, including sexual orientation and educational level, and describes how they were measured.

The corresponding changes have been incorporated into the Methods section (“Study variables” subsection) on page 7, lines 145–149.

6. The assessment of antiretroviral therapy (ART) adherence based on self-report requires further clarification. It should be specified whether this information was collected by research staff or clinical personnel, and whether it was obtained from medical records or direct patient interviews. If recorded in clinical charts, the manuscript should clarify how this information was originally collected and the extent to which it can be considered reliable, given the known limitations of self-reported adherence data.

Response: We thank the reviewer for this important observation. We clarified that ART adherence was based on patient self-report elicited by the treating clinical team during routine care and documented in the electronic medical record; this information was subsequently abstracted by the research team. No adherence interviews were conducted specifically for this study. We also clarified the operational definition used for adherence and explicitly acknowledged the limitations of self-reported adherence, including recall and social-desirability bias, which may lead to overestimation. In addition, we noted that admission HIV viral load was available as an objective complementary marker of treatment response.

The corresponding changes have been incorporated into the Methods section on page 8, lines 181–187, and the Limitations section on page 28, lines 496–499.

7. The statistical analysis section does not adequately describe the handling of missing data. The authors should report the extent of missingness for each variable, specify the methods used to handle missing data (e.g., complete-case analysis or imputation).

Response:

We thank the reviewer for this important observation. We have expanded the Statistical Analysis section to clarify the handling of missing data. No imputation was performed, and analyses were conducted using available data for each variable. Variables that were conditional by design were analyzed within their applicable subgroups and were not considered missing when not applicable. Values recorded as unknown or not documented were retained as separate categories in descriptive analyses. In addition, the extent of missingness and the corresponding analytic denominators are now explicitly reported in Table 1, with Table 2 referring to these data.

These changes have been incorporated into the Statistical Analysis section on page 9, lines 202–206; the note to Table 1 on page 16, lines 289–296; and the note to Table 2 on page 21, lines 341–342.

Results Section:

8. “In total, 2,109 records from the hospital admini

Attachment Submitted filename: Response_to_Reviewers.docx

# Peer review: Decision Letter 1

**DOI:** 10.1371/journal.pone.0357788.r003

**Authors:** Felix Bongomin

23 Aug 2026

Causes of hospitalization among people living with HIV in a tertiary-care hospital in Bogotá, Colombia, 2018–2024

PONE-D-25-67550R1

Dear Dr. Valderrama-Beltrán,

We’re pleased to inform you that your manuscript has been judged scientifically suitable for publication and will be formally accepted for publication once it meets all outstanding technical requirements.

Within one week, you’ll receive an e-mail detailing the required amendments. When these have been addressed, you’ll receive a formal acceptance letter and your manuscript will be scheduled for publication.

An invoice will be generated when your article is formally accepted. Please note, if your institution has a publishing partnership with PLOS and your article meets the relevant criteria, all or part of your publication costs will be covered. Please make sure your user information is up-to-date by logging into Editorial Manager at Editorial Manager® and clicking the ‘Update My Information' link at the top of the page. For questions related to billing, please contact billing support.

If your institution or institutions have a press office, please notify them about your upcoming paper to help maximize its impact. If they’ll be preparing press materials, please inform our press team as soon as possible -- no later than 48 hours after receiving the formal acceptance. Your manuscript will remain under strict press embargo until 2 pm Eastern Time on the date of publication. For more information, please contact onepress@plos.org.

Kind regards,

Felix Bongomin, MB ChB, Ph.D., FECMM

Academic Editor

PLOS One

Additional Editor Comments (optional):

Reviewers' comments:

Reviewer's Responses to Questions

**Comments to the Author**

Reviewer #1: All comments have been addressed

**********

2. Is the manuscript technically sound, and do the data support the conclusions?

Reviewer #1: Yes

**********

3. Has the statistical analysis been performed appropriately and rigorously?

Reviewer #1: Yes

**********

4. Have the authors made all data underlying the findings in their manuscript fully available?

The PLOS Data policy

Reviewer #1: Yes

**********

5. Is the manuscript presented in an intelligible fashion and written in standard English?

Reviewer #1: Yes

**********

Reviewer #1: Overall, the revisions satisfactorily address the concerns raised in this review. Subject to final editorial and minor language/consistency checks, I consider the manuscript substantially improved and suitable for publication.

**********

what does this mean?). If published, this will include your full peer review and any attached files.

If you choose “no”, your identity will remain anonymous but your review may still be made public.

**Do you want your identity to be public for this peer review?**  For information about this choice, including consent withdrawal, please see our Privacy Policy

Reviewer #1: No

**********

# Editor report: Acceptance letter

**DOI:** 10.1371/journal.pone.0357788.r004

**Authors:** Felix Bongomin

PONE-D-25-67550R1

PLOS One

Dear Dr. Valderrama-Beltrán,

I'm pleased to inform you that your manuscript has been deemed suitable for publication in PLOS One. Congratulations! Your manuscript is now being handed over to our production team.

At this stage, our production department will prepare your paper for publication. This includes ensuring the following:

* All references, tables, and figures are properly cited

* All relevant supporting information is included in the manuscript submission,

* There are no issues that prevent the paper from being properly typeset

You will receive further instructions from the production team, including instructions on how to review your proof when it is ready. Please keep in mind that we are working through a large volume of accepted articles, so please give us a few days to review your paper and let you know the next and final steps.

Lastly, if your institution or institutions have a press office, please let them know about your upcoming paper now to help maximize its impact. If they'll be preparing press materials, please inform our press team within the next 48 hours. Your manuscript will remain under strict press embargo until 2 pm Eastern Time on the date of publication. For more information, please contact onepress@plos.org.

You will receive an invoice from PLOS for your publication fee after your manuscript has reached the completed accept phase. If you receive an email requesting payment before acceptance or for any other service, this may be a phishing scheme. Learn how to identify phishing emails and protect your accounts at https://explore.plos.org/phishing.

If we can help with anything else, please email us at customercare@plos.org.

Thank you for submitting your work to PLOS One and supporting open access.

Kind regards,

PLOS One Editorial Office Staff

on behalf of

Dr. Felix Bongomin

Academic Editor

PLOS One
